# Guidelines for the Acute Treatment of Cerebral Edema in Neurocritical Care Patients

**DOI:** 10.1007/s12028-020-00959-7

**Published:** 2020-05-15

**Authors:** Aaron M. Cook, G. Morgan Jones, Gregory W. J. Hawryluk, Patrick Mailloux, Diane McLaughlin, Alexander Papangelou, Sophie Samuel, Sheri Tokumaru, Chitra Venkatasubramanian, Christopher Zacko, Lara L. Zimmermann, Karen Hirsch, Lori Shutter

**Affiliations:** 1 UK Healthcare, University of Kentucky College of Pharmacy, Lexington, KY USA; 2 Methodist Le Bonheur Healthcare, Memphis, TN USA; 3 University of Manitoba, Winnipeg, MB Canada; 4 Maine Medical Center, Portland, ME USA; 5 Ohio State University, Columbus, OH USA; 6 Emory University Hospital, Atlanta, GA USA; 7 Memorial Hermann-Texas Medical Center, Houston, TX USA; 8 The Daniel K. Inouye College of Pharmacy | University of Hawaii at Hilo, Honolulu, HI USA; 9 Stanford University Medical Center, Stanford, CA USA; 10 Penn State University Health Milton S. Hershey Medical Center, Hershey, PA USA; 11 University of California, Davis, Sacramento, CA USA; 12 University of Pittsburgh School of Medicine, Pittsburgh, PA USA

**Keywords:** Intracranial pressure, Neurocritical care, Osmotherapy, Hyperventilation, Hypertonic, Mannitol

## Abstract

**Background:**

Acute treatment of cerebral edema and elevated intracranial pressure is a common issue in patients with neurological injury. Practical recommendations regarding selection and monitoring of therapies for initial management of cerebral edema for optimal efficacy and safety are generally lacking. This guideline evaluates the role of hyperosmolar agents (mannitol, HTS), corticosteroids, and selected non-pharmacologic therapies in the acute treatment of cerebral edema. Clinicians must be able to select appropriate therapies for initial cerebral edema management based on available evidence while balancing efficacy and safety.

**Methods:**

The Neurocritical Care Society recruited experts in neurocritical care, nursing, and pharmacy to create a panel in 2017. The group generated 16 clinical questions related to initial management of cerebral edema in various neurological insults using the PICO format. A research librarian executed a comprehensive literature search through July 2018. The panel screened the identified articles for inclusion related to each specific PICO question and abstracted necessary information for pertinent publications. The panel used GRADE methodology to categorize the quality of evidence as high, moderate, low, or very low based on their confidence that the findings of each publication approximate the true effect of the therapy.

**Results:**

The panel generated recommendations regarding initial management of cerebral edema in neurocritical care patients with subarachnoid hemorrhage, traumatic brain injury, acute ischemic stroke, intracerebral hemorrhage, bacterial meningitis, and hepatic encephalopathy.

**Conclusion:**

The available evidence suggests hyperosmolar therapy may be helpful in reducing ICP elevations or cerebral edema in patients with SAH, TBI, AIS, ICH, and HE, although neurological outcomes do not appear to be affected. Corticosteroids appear to be helpful in reducing cerebral edema in patients with bacterial meningitis, but not ICH. Differences in therapeutic response and safety may exist between HTS and mannitol. The use of these agents in these critical clinical situations merits close monitoring for adverse effects. There is a dire need for high-quality research to better inform clinicians of the best options for individualized care of patients with cerebral edema.

**Electronic supplementary material:**

The online version of this article (10.1007/s12028-020-00959-7) contains supplementary material, which is available to authorized users.

## Introduction

Cerebral edema is a non-specific pathological swelling of the brain that may develop in a focal or diffuse pattern after any type of neurological injury. The underlying cause of this brain swelling is highly variable and relates to multiple physiological cellular changes. The simplest description of cerebral edema is an accumulation of excessive fluid within either brain cells or extracellular spaces. Cerebral edema can be secondary to disruption of the blood brain barrier, local inflammation, vascular changes, or altered cellular metabolism. The identification and treatment of cerebral edema is central to the management of critical intracranial pathologies. Measurement of cerebral edema is indirect and generally relies on surrogate markers seen on imaging studies, such as tissue shifts or structural changes, or via intracranial pressure (ICP) monitoring devices. It is considered one of the more common contributors to elevated ICP, which has been identified as a predictor of poor outcome in patients with TBI, stroke, and other intracranial pathologies [1, 2]. The literature describes multiple treatment modalities including hyperosmolar therapy, acute hyperventilation, temperature modulation, diversion of CSF, surgical decompression, and metabolic suppression [3]. These treatments are often administered without consideration of the underlying disease process, when in fact their efficacy may hinge upon the pathophysiology at hand. Recent guidelines for the management of AIS, ICH, and TBI, among others, discuss the treatment of cerebral edema. However, practical recommendations regarding the selection and monitoring of therapies for optimal efficacy and safety are generally lacking [3–6].

This guideline primarily evaluates the role of hyperosmolar agents (mannitol, HTS), corticosteroids, and selected non-pharmacologic therapies in the acute treatment of cerebral edema; strategies used for refractory cerebral edema or increased ICP (e.g., barbiturates, therapeutic hypothermia) are not highlighted. The term cerebral edema was used preferentially as a representative term encompassing elevated intracranial pressure, brain swelling, herniation syndromes, and cerebral edema (intracranial pressure may not be known in many patients, but symptoms of this intracranial abnormality may be present). In references where intracranial pressure was specifically evaluated, the results are stated as such. It should be emphasized that the recommendations in this guideline are based on available medical literature, which may not reflect all aspects of clinical expertise and practical experience.

## Methods

This work was commissioned and approved by the Neurocritical Care Society (NCS) Board of Directors. The NCS Guidelines Committee tasked two Chairs (AC, LS) to form a panel of experts in neurocritical care, pharmacotherapy, and nursing to execute the guideline. A GRADE methodologist was also included in the panel. Beginning in October 2017, the panel drafted sixteen questions relevant to the treatment and monitoring of cerebral edema in neurocritical care patients using the Grading of Recommendations Assessment, Development, and Evaluation (GRADE) framework (Table 1) [7]. Questions were drafted using the PICO format and aligned with specific neurocritical care populations (e.g., TBI) so as to render recommendations relevant to underlying pathologies and not the neurocritical care population as a whole. Individual outcomes of interest related to cerebral edema, ICP, neurological status, or other evaluated parameters are listed on the applicable tables that are included as electronic supplementary material. Of note, the response of intracranial pressure or cerebral edema was separated from neurological outcomes such as modified Rankin Score or mortality as the authors were uncertain that treatment response of intracranial pressure or cerebral edema is directly related to neurological outcome. Interventions were specifically identified by name and formulation in most instances. In some PICO questions, various sodium salts with differing concentrations were evaluated. Throughout the document, “hypertonic saline” was referred to as “hypertonic sodium solutions” to account for the differences in sodium salt formulation. In cases where a specific sodium salt or concentration was evaluated, it was identified as stated in the study if the specific formulation was pertinent for the results.

A research librarian executed a comprehensive, independent literature search using Ovid Medline and EMBASE from inception (1947) to July 5, 2018. The searches were peer reviewed by a second librarian at St. Michael’s Hospital in Toronto, ON. Additional articles were identified from the bibliographies of the publications included in the literature search, as well as a supplementary PubMed searches of publications and personal files of members of the panel up to February 2019 once data abstraction began. The literature search excluded articles that were not available in English, pediatric studies, animal studies, and unpublished works. The panel included systematic reviews, meta-analyses, randomized, controlled trials, observational studies, and case series of five or more patients. Studies with prospectively collected data which were retrospectively evaluated were consistently considered retrospective observational studies in our quality of evidence assessment. Studies with mixed populations (e.g., SAH, TBI, and ICH) were included if the population relevant to the specific PICO question had a sample size of five or more. Meta-analyses were included only if their patients were representative of the population of neurocritical care patients identified in the specific PICO question. If the meta-analysis included a mixture of multiple populations, the results were only included if they were differentiated by neurological injury. All meta-analyses that were excluded due to an inability to differentiate results by specific neurological injury are listed as footnotes on the applicable evidentiary tables that are included as electronic supplementary material. A systematic review software was used for screening and abstraction of the available literature (DistillerSR, Evidence Partners, Ottawa, Canada). Initial article screening for inclusion criteria was performed by one individual reviewer. Once screened for inclusion, data abstraction from each of the pertinent articles was performed by a minimum of two panel members pertaining to each PICO question (Table 1).

The panel used the GRADE methodology to evaluate the quality of evidence as high, moderate, low, or very low [7]. These designations denote the degree of certainty that the estimate of effect in each study approximates the true effect. Recommendations generated from this literature review and the subsequent quality of evidence rating accounted for efficacy, risks, potential sources of bias, and treatment effect. The Cochrane Risk of Bias Assessment tool was used to evaluate bias in sequence generation, allocation, blinding, missing outcome data, selective outcome reporting, and other sources of bias. The panel classified recommendations as strong (“We recommend”) when they are the preferred treatment for most patients and should be adopted as policy in the majority of situations. Conditional recommendations (“We suggest,” “Clinicians should consider”) should be further considered based upon the clinical scenario and carefully evaluated by stakeholders before being implemented as policy. Areas where there is insufficient evidence to support recommendations are identified, and, in some instances, “good practice statements” are provided. These statements are meant to state guidance where there may be a lack of published evidence, but the practice is commonly accepted as beneficial.

The panel met in person on September 26–27, 2018, in Boca Raton, FL, and again on January 17–18, 2019, in Chicago, IL. Other meetings were virtual. Members assigned to specific PICO questions presented a summary of the GRADE evidence on each topic, and recommendations were discussed, revised, and validated by the entire panel. Internal experts within the Neurocritical Care Society and external stakeholders reviewed the final guideline.

### Treatment of Cerebral Edema in Patients with Subarachnoid Hemorrhage

- In patients with SAH, does sodium target-based dosing with hypertonic sodium solutions (sodium chloride, lactate, or bicarbonate) improve ICP/cerebral edema compared to intermittent, symptom-based bolus doses of hypertonic sodium solutions?
- In patients with SAH, does sodium target-based dosing with hypertonic sodium solutions (sodium chloride, lactate, or bicarbonate) improve neurological outcomes at discharge compared to intermittent, symptom-based bolus doses of hypertonic sodium solutions?

#### Recommendations

1. We suggest using symptom-based bolus dosing of hypertonic sodium solutions rather than sodium target-based dosing for the management of ICP or cerebral edema in patients with SAH (conditional recommendation, very low-quality evidence).

##### *Rationale*:

In making this recommendation, the panel felt that while the quality of evidence was very low, the consistency of the literature justified symptom-based bolus dosing of HTS as an effective means of reducing ICP and cerebral edema in patients with SAH. The data on sodium target-based HTS dosing regimens for ICP control were extremely limited and provided only indirect evidence and thus could not be recommended.

2. Due to insufficient evidence, we cannot recommend a specific dosing strategy for HTS to improve neurological outcomes in patients with SAH.

The panel first evaluated the relative merits of symptom-based bolus dosing of HTS as opposed to HTS administration titrated to a target sodium concentration in patients with SAH (Table 1, Questions 1 and 2). A small number of publications have addressed this specific issue, and the panel did not identify any studies that directly compared the two administration strategies. Nine studies addressed the two infusion strategies independently: two targeted a sodium level of 145–155 mEq/L and seven used symptom-based bolus administration of HTS [8–16]. The overall quality of evidence was very low (Table 2).

The panel assessed seven studies of symptom-based bolus administration of HTS. One key study for many of the topics in this guideline is Koenig, et al., which was a retrospective cohort analysis that evaluated the percentage of neurocritical care patients who experienced clinical reversal of TTH after receiving a 30–60 mL dose of 23.4% NaCl [10]. The investigators observed clinical TTH reversal in 75% of cases and 22 patients (32.4%) survived to hospital discharge. Only 16 of 68 patients included had SAH and the results were only reported for the full cohort (i.e., not subdivided to show the SAH patient data specifically), so the direct effects on patients with SAH could not be discerned. Overall, reversal of TTH was associated with a > 5 mEq/L rise in serum sodium concentration (p < 0.001) or an absolute serum sodium of > 145 mEq/L (p < 0.007) within 1 h after 23.4% HTS administration. It should be noted that while changes in sodium values were observed, specific targets had not been pre-determined.

In a prospective study, Al-Rawi et al. administered 2 ml/kg of 23.5% NaCl to 44 patients with poor-grade SAH [14]. Patients who demonstrated a robust and durable response to the NaCl infusion were more likely to have a favorable outcome (mRS < 4). Bentsen and colleagues published three studies using 7.2% NaCl with 6% HES, a formulation which is currently not available in the USA, in patients with SAH [11–13]. One of these was a prospective, randomized, placebo-controlled trial of 22 patients that found modest reductions in ICP using 7.2% HTS/6% HES compared to placebo [11]. The other studies were of lower quality with similar results [12, 13].

Two studies explored the effects of titrating HTS to a targeted serum sodium concentration of 145–155 mEq/L. Tseng, et al. prospectively observed 35 patients who received 2 ml/kg doses of 23.5% NaCl [16]. They concluded that HTS administration in this manner decreased ICP and improved CBF, though their results were obtained via logistic regression analysis of dose-dependent effects of HTS on CBF as measured by CT scan perfusion studies. The panel deemed that these measures were too indirect to be considered outcomes as defined by the PICO question, which downgraded the rating of this study. A second study retrospectively compared patients who received a 3% NaCl continuous infusion titrated to a goal serum sodium of 145–155 mEq/L to a group of historical controls who did not receive HTS [9]. While the total sample size was robust (n = 215), only 38 patients had SAH. This subgroup was underpowered, and there was not a statistically significant difference in episodes of critically elevated ICP or neurological outcomes. Given both studies were underpowered and used different administration methods to achieve the target sodium concentration, the panel felt the available data prevented endorsement of HTS use to target a specific serum sodium concentration in order to improve ICP, cerebral edema, or neurological outcomes.

While the overall quality of the evidence in this area is very low, the panel felt there was enough consistency across the published studies to suggest symptom-based bolus dosing of HTS as an effective means of reducing ICP and cerebral edema in patients with SAH. In addition, HTS bolus administration may also raise serum sodium, improve brain pH, and increase brain tissue oxygenation [17, 18]. At present, there are insufficient data to support the use of HTS to improve neurological outcome, regardless of the administration strategy. We did not identify any substantial evidence to support targeting a specific serum sodium concentration in order to reduce ICP or improve neurological outcome in patients with SAH, demonstrating the need for future research regarding the impact of specific sodium targets on ICP and neurological outcomes.

### Treatment of Cerebral Edema in Patients with Traumatic Brain Injury

- In patients with TBI, does the use of hypertonic sodium solutions improve cerebral edema compared to mannitol?
- In patients with TBI, does the use of hypertonic sodium solutions for cerebral edema improve neurological outcomes compared to mannitol?

#### Recommendations

1. We suggest using hypertonic sodium solutions over mannitol for the initial management of elevated ICP or cerebral edema in patients with TBI (conditional recommendation, low-quality evidence). We suggest that neither HTS nor mannitol be used with the expectation for improving neurological outcomes in patients with TBI (conditional recommendation, low-quality evidence).

##### *Rationale*:

In making this recommendation, the panel felt that while the quality of evidence was low, the consistency of the literature suggested HTS was at least as safe and effective as mannitol. In addition, the panel agreed that the putative advantages of HTS over mannitol for fluid resuscitation and cerebral perfusion justified the suggestion to use HTS over mannitol. Although treatment effect of these agents on elevated ICP or cerebral edema may be expected based on the literature, neither agent has been demonstrated to improve neurological outcomes.

2. We suggest that the use of mannitol is an effective alternative in patients with TBI unable to receive hypertonic sodium solutions (conditional recommendation, low-quality evidence).

##### *Rationale*:

Although HTS was recommended over mannitol, the quality of evidence was low and the literature consistently suggests that mannitol is also a safe and effective option for the initial management of elevated ICP or cerebral edema in patients with TBI, particularly those with concomitant severe hypernatremia or volume overload.

3. We recommend against the use of hypertonic sodium solutions in the pre-hospital setting to specifically improve neurological outcomes for patients with TBI (strong recommendation, moderate-quality evidence).

##### *Rationale*:

Acute treatment of cerebral edema and herniation syndromes is often necessary in the pre-hospital setting. However, well-designed clinical trials did not suggest any benefit from the use of HTS in the pre-hospital setting on long-term outcomes in patients with TBI.

4. We suggest against the use of mannitol in the pre-hospital setting to improve neurological outcomes for patients with TBI (conditional recommendation, very low-quality evidence).

##### *Rationale*:

In making this recommendation, the panel felt that the quality of evidence was very low and did not suggest any potential benefit in the pre-hospital setting on long-term outcomes in patients with TBI.

The panel evaluated several studies to inform recommendations on the use of HTS and/or mannitol to improve ICP, cerebral edema, or neurological outcomes (Table 1, Questions 3 and 4). These two agents have been compared in at least eight randomized, controlled trials of patients with elevated ICP from a variety of causes, including TBI [19–26]. A number of uncontrolled, retrospective, and non-comparative studies have also evaluated either HTS or mannitol in patients with TBI. These studies all support the notion that both hyperosmolar therapies effectively reduce ICP (Table 3). The available data are limited by patient heterogeneity, low sample size, and inconsistent methods among studies. For example, several studies utilized a crossover study design where patients served as their own control, sequentially receiving HTS and then mannitol or vice versa, whereas others randomized patients to receive one agent or the other [20, 22]. In addition, several of the early studies evaluating HTS used a combination product which included HES. The overall quality of evidence was low (Evidentiary Table 2 of Electronic Supplementary Material).

The panel discussed at length the lack of high-quality evidence supporting preferential use of one hyperosmolar agent as first line treatment of elevated ICP in TBI. Several meta-analyses have reached conflicting conclusions; some found no difference in ICP-related outcomes in TBI patients, whereas others favored HTS [27, 28]. Differences in the statistical analysis across these meta-analyses may have accounted for the variance in results [28].

Some advantages of HTS over mannitol have been observed in direct comparison, crossover, and rescue therapy randomized studies [28]. HTS may have a quicker onset of action, a more robust and durable ICP reduction, and may be advantageous in patients in whom mannitol failed [21]. Hypertonic sodium solutions with either a chloride, lactate, or bicarbonate salt seem to all be effective [26, 29]. The panel felt that there was consistency across the numerous, lower-quality studies that HTS was more effective than mannitol for reducing ICP or cerebral edema in this population.

Several meta-analyses and RCTs have demonstrated no significant difference in neurological outcomes when comparing various hyperosmolar therapies [19, 24, 27, 30]. It is notable that two of the RCTs used equi-osmolar doses of HTS and mannitol, whereas the third used twofold higher osmolar dose of 7.5% NaCl compared to mannitol [19, 24, 30]. Another prospective study pooled data from three separate trials and evaluated target-based infusions of 20% NaCl compared to standard care [31]. Overall there was no difference in long-term neurological outcomes between groups, but patients who received 20% NaCl exhibited a reduced mortality rate at 90 days in a propensity-score-adjusted analysis (HR 1.74, 95% CI 1.36–2.23). Nevertheless, the weight of the current evidence does not support the use of hyperosmolar therapies for the specific purpose of improving neurological outcomes.

Studies have also evaluated emergent, pre-hospital resuscitation with HTS or mannitol in patients with TBI. A phase II feasibility study evaluated 229 TBI patients with a GCS < 9 and those who were hypotensive (SBP < 100 mm Hg) and randomized each to receive pre-hospital 250 mL 7.5% NaCl or 250 mL Ringer’s lactate. Survival to hospital discharge was similar in both groups, as was GOSE and survival at 6 months [32]. Bulger et al. performed a prospective, double-blind trial of 1282 TBI patients who received a 250 mL bolus of 7.5% NaCl/6% dextran 70, a 250 mL bolus of 7.5% NaCl, or a 250 mL 0.9% NaCl in the pre-hospital setting [33]. No significant differences in distribution of GOSE category, Disability Rating Score, or mortality by treatment group were found. Sayre et al. evaluated the use of mannitol in the pre-hospital setting in patients with TBI and also found no benefit on mortality [34].

While the overall quality of the evidence in this area is low, the panel felt there was enough consistency across the published studies to suggest that both HTS and mannitol are effective in reducing ICP elevations and cerebral edema. The panel noted that the evidence supporting HTS is more robust, but mannitol is also an effective option. The decision regarding which agent to use should be based on available resources, the patient’s individual characteristics, and local practice patterns. While either agent can address the physiological abnormalities of ICP elevations and cerebral edema, there is evidence that neither agent directly influences long-term neurological outcome, particularly when used in the pre-hospital setting.

### Treatment of Cerebral Edema in Patients with Acute Ischemic Stroke

- In patients with ischemic stroke, does the use of hypertonic sodium solutions improve cerebral edema compared to mannitol?
- In patients with ischemic stroke, does the use of hypertonic sodium solutions for cerebral edema improve neurological outcomes compared to mannitol?

#### Recommendations

1. We suggest using either hypertonic sodium solutions or mannitol for the initial management of ICP or cerebral edema in patients with acute ischemic stroke (conditional recommendation, low-quality evidence). There is insufficient evidence to recommend either hypertonic saline or mannitol for improving neurological outcomes in patients with acute ischemic stroke.

##### *Rationale*:

In making this recommendation, the panel felt that the quality of evidence was low and the literature in patients with AIS was not compelling to recommend one agent over the other for initial management of elevated ICP or cerebral edema. Patient-specific factors may be employed to aid clinicians in selecting the appropriate initial agent in patients with either measured elevated ICP or symptoms of cerebral edema.

2. We suggest that clinicians consider administration of hypertonic sodium solutions for management of ICP or cerebral edema in patients with acute ischemic stroke who do not have an adequate response to mannitol (conditional recommendation, low-quality evidence).

##### *Rationale*:

In making this recommendation, the panel felt that the quality of evidence was low, but the literature in patients with AIS suggested that patients who do not have an adequate treatment response to mannitol may still respond to HTS.

3. We suggest against the use of prophylactic scheduled mannitol in acute ischemic stroke due to the potential for harm (conditional recommendation, low-quality evidence).

##### *Rationale*:

In making this recommendation, the panel felt that while the quality of evidence was low, the lack of benefit and the potential association with worse neurological outcomes justified avoiding prophylactic mannitol in patients with acute ischemic stroke.

The panel evaluated whether the use of HTS or mannitol improves ICP, cerebral edema, or neurological outcomes in patients with AIS. The panel did not identify any RCTs that directly compared these agents in AIS using neurological outcomes as an endpoint. Therefore, we included observational studies that either compared mannitol or HTS with no hyperosmolar therapy or that reported on hyperosmolar therapy use without a control group (Table 1, Questions 5 and 6). The overall level of evidence was low (Table 4).

The panel identified 11 studies that evaluated hyperosmolar therapy for reducing ICP or cerebral edema in patients with AIS. Of these, three were prospective, randomized trials that compared mannitol directly with HTS [21, 35, 36]. The remainder were prospective or retrospective cohort studies that evaluated one or both hyperosmolar therapies with or without a control group. The majority of these studies suggested that both HTS and mannitol were efficacious in reducing ICP in AIS, although some of these studies had a mixed patient population [9, 21, 36–39]. In two prospective, randomized studies, the reduction in ICP from HTS was quicker, more pronounced, and more sustained compared to mannitol [21, 36]. In addition, two studies suggested that HTS may be effective even in patients in whom mannitol has failed [36, 40].

While the use of a continuous infusion of HTS titrated to sodium goals is a common practice in neurocritical care, use of this strategy in AIS has only been evaluated in two studies which had conflicting results. One evaluation suggested that the use of continuous HTS to target a serum sodium concentration of 145–155 mEq/L may be associated with fewer ICP crises per patient, while the other demonstrated no difference [9, 37]. Based on the available evidence, the use of 3% NaCl to target a specific serum sodium concentration does not consistently demonstrate reductions in ICP crises and does not appear to improve neurological outcome in patients with AIS.

Overall, both mannitol and HTS appear to be effective in reducing ICP and cerebral edema in patients with AIS. Given the limitations of the above studies (small sample size, heterogeneous populations, insufficient information on osmolar load), we cannot definitively conclude that one hyperosmolar therapy agent is clearly superior to the other for ICP reduction. However, it appears HTS may have a more rapid onset of action, a more robust and durable ICP reduction, and may be advantageous for patients in whom mannitol failed [36, 40].

After having established the potential benefits of hyperosmolar therapy in improving intracranial pressure, the panel next evaluated 10 studies that focused on hyperosmolar therapy to improve neurological outcome in AIS. Of these studies, only one was an RCT (which compared mannitol to no hyperosmolar therapy) [41]. Only two other studies classified outcomes separately between mannitol and HTS in AIS; however, these studies did not directly compare these treatments and therefore an analysis of the relative efficacy was not possible [42, 43]. Of these studies, hyperosmolar therapies were administered in a number of different ways, ranging from scheduled daily dosing, dosing to a specific serum sodium target (in the case of HTS), or intermittent dosing over the period of several days [9, 10, 37, 41, 42, 44–47]. The indication for hyperosmolar therapy was also variable: ICP elevation, cerebral edema, or prophylactic [10, 37].

Overall, the data are weak and contradictory when evaluating the effect of hyperosmolar agents on neurological outcome after AIS. An analysis of a prospective registry of patients reported improved mortality and 3-month mRS in AIS treated with 5.1–7.6% NaCl, but this was not compared with another therapy or control group [42]. Another small case series reported improvement in GCS or pupillary reactivity in some patients after a single mannitol dose, but, with only seven patients and no control group, definitive conclusions are not possible [44]. The Koenig study was also included to address this PICO question, but the potential benefit of 23.4% NaCl in AIS patients is not discernable from this report [10]. The only RCT is an outdated study of 77 patients with AIS which found no change in neurological outcome after a single daily dose of 0.8 to 0.9 g/kg mannitol for 10 days [41]. The majority of the observational studies also reported no difference in neurological outcome after hyperosmolar therapy administration [9, 45, 47, 48].

Conversely, some reports have suggested harm with prophylactic hyperosmolar therapy with mannitol after AIS. Two large, retrospective, cohort studies reported an increased risk of death at 30 days and/or greater functional dependency with prophylactic use of scheduled mannitol doses [45, 46]. However, the findings of both of these studies may have been skewed by the potential for dosing mannitol more frequently in the most critically ill patients. This potential treatment bias could not be fully accounted for after multivariable adjustment.

While the overall quality of evidence in this area is low, the panel felt there was enough consistency across published studies to suggest that both HTS and mannitol are effective in reducing ICP elevations and cerebral edema in AIS. In contrast, the overall evidence does not support routine hyperosmolar therapy to improve neurological outcome following AIS, and in fact, the use of prophylactic mannitol may be associated with harm.

### Treatment of Cerebral Edema in Patients with Intracerebral Hemorrhage

- In patients with ICH, does the use of hypertonic sodium solutions improve cerebral edema compared to mannitol?
- In patients with ICH, does the use of corticosteroid therapy improve neurological outcomes compared to placebo/control?

#### Recommendations for Hyperosmolar Therapy

1. We suggest using hypertonic sodium solutions over mannitol for the management of ICP or cerebral edema in patients with intracerebral hemorrhage (conditional recommendation, very low-quality evidence).

##### *Rationale*:

In making this recommendation, the panel felt that while the quality of evidence was very low, the consistency of the literature suggested HTS was at least as safe and effective as mannitol. In addition, the panel agreed that the putative advantages of HTS over mannitol for fluid resuscitation and cerebral perfusion justified the suggestion to use HTS over mannitol.

2. We suggest that either symptom-based bolus dosing or using a targeted sodium concentration is appropriate hypertonic sodium solution administration strategy for the management of elevated ICP or cerebral edema in patients with intracerebral hemorrhage (conditional recommendation, very low-quality evidence).

##### *Rationale*:

In making this recommendation, the panel felt that the quality of evidence was very low and the literature in patients with ICH was not compelling to recommend one method of administration of HTS over the other for initial management of elevated ICP or cerebral edema. Patient-specific factors may be employed to aid clinicians in selecting the appropriate initial agent.

The panel evaluated the relative merits of HTS and mannitol in improving ICP, cerebral edema, or neurological outcomes in patients with ICH (Table 1, Question 7). The panel identified four studies examining HTS alone, one related to mannitol, and no studies comparing the two agents. The overall quality of evidence was very low (Table 5).

Three studies addressed the use of continuous infusion 3% NaCl adjusted to achieve a targeted sodium concentration of 145–155 mEq/L [9, 37, 49]. Hauer et al. reported on a mixed neurocritical care patient population that included 120 patients with ICH [9]. These patients received 3% NaCl infusions with a goal serum sodium of 145–155 mEq/L compared to a historical cohort, and the results are reported specific to patients with ICH. A prospective study of 26 ICH patients treated with 3% NaCl continuous infusion demonstrated a reduction in cerebral edema and number of ICP crises [49]. Conversely, a retrospective cohort study with a subset of eight ICH patients who received 3% sodium acetate/chloride continuous infusion (with additional boluses as needed) found no differences in ICP or mass effect at 12 h [37]. Data from the aforementioned Koenig study was included to address this PICO question as patients with ICH were included even though they could not be separated out from the other patient populations [10]. The data from these studies are conflicting, and clinicians should weigh the risks and benefits of targeting a sodium concentration in the short-term management of ICP and cerebral edema in ICH patients.

The panel acknowledges that there are some commonly referenced articles reporting on the use of mannitol in patients with ICH that were not included in this guideline, as they did not address these specific PICO questions [50–52]. Two recent publications, both of which demonstrated potential risk of hematoma expansion, were ultimately excluded from our assessment as the study outcomes did not directly address the PICO questions as written (though hematoma expansion is important clinically, the panel did not feel this variable was directly related to cerebral edema or ICP) [53, 54]. However, the panel felt that their results may give providers some pause regarding the safety of mannitol in patients with ICH. Given these concerns, along with the lack of published articles assessing the impact of mannitol on short-term outcomes, the panel was unable to recommend use of mannitol in this population at this time.

While the overall quality of evidence in this area is very low, the panel felt there was enough consistency across published studies to suggest that HTS is effective in reducing ICP elevations and cerebral edema. In contrast, the available published studies on mannitol were not directly related to the PICO question and they implied harm; thus, the panel favored HTS over mannitol.

#### Recommendations for Corticosteroids in Patients with Intracerebral Hemorrhage

1. We recommend against the use of corticosteroids to improve neurological outcome in patients with intracerebral hemorrhage due to the potential for increased mortality and infectious complications (strong recommendation, moderate-quality evidence).

The panel evaluated whether use of corticosteroids in patients with ICH impacts neurological outcome, including mortality or functional status at any designated time point. The panel included a Cochrane meta-analysis, two moderate- to high-quality studies, and several other lower-quality studies in their assessment (Table 1, Question 8) [55–62]. The overall quality of evidence was moderate (Table 6).

A 2005 Cochrane Review assessed the impact of corticosteroids on neurological outcome in patients with ICH compared to placebo or standard of care controls. The review found no evidence to support the routine use of corticosteroids in patients with primary ICH and highlighted a potential for harm in these patients [61]. One prospective, randomized, placebo-controlled trial (n = 93) of dexamethasone to placebo in patients with ICH was halted due to increased rates of infectious and diabetic complications, while another (n = 40) showed no difference in mortality or neurological outcomes [55, 62]. Sharafadinzadeh et al. published a placebo-controlled trial of 225 patients which found higher mortality in the dexamethasone group [56]. Four other lower-quality studies all found no improvement in outcomes related to dexamethasone use, with two studies demonstrating increased mortality in the treatment group [57–60].

### Treatment of Cerebral Edema in Patients with Bacterial Meningitis

- In patients with bacterial meningitis, does the use of hypertonic sodium solutions for cerebral edema improve CE compared to mannitol?
- In patients with bacterial meningitis, does the use of corticosteroid therapy improve neurological outcomes compared to placebo/control?

#### Recommendations

1. We recommend dexamethasone 10 mg intravenous every 6 h for 4 days to reduce neurological sequelae (primarily hearing loss) in patients with community-acquired bacterial meningitis (strong recommendation, moderate-quality evidence).
2. We suggest dexamethasone 0.15 mg/kg intravenous every 6 h for 4 days as an alternative dose for patients with low body weight or high risk of corticosteroid adverse effects (good practice statement).
3. We recommend administering dexamethasone before or with the first dose of antibiotic in patients with bacterial meningitis (strong recommendation, moderate-quality evidence).
4. We recommend use of corticosteroids to reduce mortality in patients with tuberculosis meningitis (strong recommendation, moderate quality of evidence). We cannot make a recommendation for one specific corticosteroid or dose in patients with TB meningitis due to the inconsistency of agents and doses evaluated in the literature.
5. We suggest that treatment with corticosteroids should be continued for two or more weeks in patients with tuberculosis meningitis (conditional recommendation, low quality of evidence).

##### *Rationale*:

In making this recommendation, the panel felt that the quality of evidence was low and considerable variability exists within and across studies regarding the duration of corticosteroid treatment. Clinicians should use patient-specific response and clinical factors to optimize the duration of corticosteroid treatment in this setting.

6. There is insufficient evidence to determine whether hypertonic sodium solutions or mannitol is more effective to reduce ICP or cerebral edema in patients with community-acquired bacterial meningitis.

The panel evaluated whether the use of corticosteroids improved neurological outcome in patients with community-acquired bacterial meningitis and TB meningitis. (Table 1, Questions 9 and 10) The panel elected to focus on these two areas within the broader category of bacterial meningitis due to their acuity and worldwide prevalence. The literature describing corticosteroid use in community-acquired bacterial meningitis is broad and includes many studies that combined adult and pediatric populations, as well as mixed immunocompetent, immunocompromised, and unknown immune status patients. While various corticosteroids have been evaluated, the overwhelming majority administered dexamethasone. Specific antimicrobial use and the corticosteroid timing in relation to antibiotic dose were not always described but are noted in the applicable evidentiary table when the information was available. The overall quality of the evidence was moderate (Tables 7 and 8).

#### Bacterial Meningitis

Three meta-analyses have shown varying effects of corticosteroid treatment on outcomes in community-acquired bacterial meningitis [63–65]. These meta-analyses are disparate in their methodologies and assess patients over different time periods, but are similar in that they included several of the same randomized trials. Due to the high risk of heterogeneity across these meta-analyses and the low quality of some of the studies included in each meta-analysis, the overall quality of these meta-analyses was classified as low and the strength of these findings should be cautiously applied in practice. In addition, some overlap in the data and time periods exists; therefore, the panel took this into account so as to not over-emphasize specific studies in the recommendations. One meta-analysis by Brouwer et al. included 25 RCTs from 1963–2013 and evaluated children and adults with varying corticosteroid agents and doses. Overall, there were no significant differences in mortality or neurological sequelae, although corticosteroid use significantly lowered rates of hearing loss [63]. Patients in high-resource areas who were treated with corticosteroids had decreased hearing loss compared to those in low-resource areas. In addition, a subset of patients with *Streptococcus pneumoniae* meningitis demonstrated a lower mortality rate with corticosteroid treatment [63]. When seven studies from this meta-analysis were analyzed separately with only adult patient data, there was no difference in mortality between groups. Four of these studies included hearing loss as part of their neurological outcome measures and found it to be significantly lower in patients treated with corticosteroids.

A second meta-analysis by Vardakas et al. included 10 RCTs published from 1963 to 2007 and determined that corticosteroids were not associated with decreased mortality overall [64]. A subgroup analysis found that corticosteroid treatment was associated with lower mortality in laboratory-proven meningitis, *Streptococcus pneumoniae* meningitis, and in patients in high-resource areas. Further analysis of only the high-quality randomized controlled trials in the meta-analysis showed that corticosteroid use was associated with decreased hearing loss. An additional subgroup analysis removing primarily HIV-positive patients demonstrated that corticosteroid treatment was associated with lower mortality [64, 66]. Finally, Van de Beek et al. published a meta-analysis of five trials published between 2002 and 2007 using individual patient data [67]. Overall, they found no difference in mortality, neurological disability, or severe hearing loss. A post hoc analysis suggested that, among survivors, corticosteroid use was associated with reduced hearing loss.

Individual trials show similar trends. de Gans et al. published the landmark RCT for corticosteroid use in community-acquired bacterial meningitis, which randomized adult patients to dexamethasone 10 mg intravenous every 6 h for 4 days with the first dose of dexamethasone 15–20 min prior to or with antibiotics [68]. Dexamethasone improved GOS and decreased mortality, but did not benefit other neurological sequelae including hearing loss. In a subgroup analysis of patients with *Streptococcus pneumoniae* meningitis, neurological outcomes and the rate of neurological sequelae were improved in the dexamethasone group compared with placebo. Other studies have also suggested patients with *Streptococcus pneumoniae* meningitis may exhibit reduced hearing loss and lower risk of mortality from corticosteroid therapy [63, 64, 69, 70]. Taken together, these three meta-analyses and multiple individual trials suggest that corticosteroids do not affect mortality overall, though some distinct patient subsets may gain mortality benefit. In addition, corticosteroids may play a role in mitigating hearing loss.

Of note, the Infectious Disease Society of American Bacterial Meningitis Guidelines evaluated corticosteroid use in 2004 [71]. These guidelines included both adult and pediatric patients. The authors recommended use of dexamethasone in adults with suspected or proven *Streptococcus pneumoniae* meningitis and avoiding corticosteroid use if antimicrobials have already been initiated (both with a GRADE-equivalent to strong recommendation, high-quality evidence). They also noted that the data are inadequate to recommend dexamethasone in adults with meningitis due to an unknown or non-pneumococcal pathogen. The panel elected to recommend the dexamethasone dose used in the de Gans study as it was the seminal study and provides an easy, practical dosing regimen.

There is a scarcity of the published literature addressing the use of HTS or mannitol for ICP elevation or cerebral edema in community-acquired bacterial meningitis (Table 9). The panel identified one relevant study, which did not specifically evaluate the role of hyperosmolar therapy but rather determined that ICP control by a combination of methods improved mortality in community-acquired bacterial meningitis [72]. Of note, only 40% of patients included in this study received hyperosmolar therapy. Multiple studies evaluate the use of glycerol for elevated ICP in community-acquired bacterial meningitis and did not show a mortality benefit [73, 74].

#### Tuberculous Meningitis

The panel evaluated the use of corticosteroid treatment in patients with TB meningitis separately from community-acquired bacterial meningitis (Table 1, Questions 9 and 10). Tuberculous meningitis occurs in immunocompromised patients, is more frequent in lower-resource areas, and generally has a higher morbidity and mortality compared to community-acquired bacterial meningitis [75]. The literature search identified a number of RCTs and one meta-analysis, as well as a number of other studies evaluating corticosteroids in TB meningitis. The overall quality of the evidence was moderate (Evidentiary Table 8 of Electronic Supplementary Material).

The meta-analysis by Prasad et al. included 1337 adult and pediatric patients with TB meningitis who received corticosteroids. Studies using a variety of agents and over different durations were also included in this analysis [76]. At 3–18-month follow-up, corticosteroid use reduced mortality by almost 25%, but did not change overall neurological outcomes. These results are consistent with an RCT of moderate quality that evaluated a tapering dose of dexamethasone and found that it reduced mortality, but not neurological deficits. This treatment effect was consistent across subgroups of disease severity and HIV status [77]. Conversely, other randomized trials in TB meningitis have shown no benefit on mortality or morbidity and were confounded by a variety of corticosteroid dosing strategies and agents used across the studies. The duration of corticosteroid therapy ranged from 1 to 8 weeks in these studies, and no definitive treatment duration has emerged from the available evidence [78–80]. Overall, there is some suggestion that corticosteroids may reduce mortality in patients with tubercular meningitis and the duration of therapy is likely to be much longer than with community-acquired meningitis.

### Treatment of Cerebral Edema in Patients with Hepatic Encephalopathy

- In patients with hepatic encephalopathy, does the use of hypertonic sodium solutions improve cerebral edema compared to mannitol?
- In patients with hepatic encephalopathy, does the use of hyperosmolar therapy improve neurological outcomes at discharge compared to ammonia-lowering agents?

#### Recommendations

1. We suggest using either hypertonic sodium solutions or mannitol for the management of ICP or cerebral edema in patients with hepatic encephalopathy (conditional recommendation, very low-quality evidence).

##### *Rationale*:

In making this recommendation, the panel felt that the quality of evidence was very low and the literature in patients with hepatic encephalopathy was not compelling to recommend one form hyperosmolar therapy over the other. Thus, either agent could be used and patient-specific factors may be employed to aid clinicians in selecting the appropriate initial agent.

2. There is insufficient evidence to determine whether either hyperosmolar therapy or ammonia-lowering therapy improves neurological outcomes in patients with hepatic encephalopathy.

The panel assessed whether the use of HTS reduced ICP or cerebral edema more effectively than mannitol in patients with HE (Table 1, Question 11). The panel identified five studies that evaluated either mannitol or HTS as monotherapy, but did not find any studies that directly compared the two agents [81–85]. The overall quality of evidence was very low (Table 10).

Early studies evaluated various combinations of therapies (mannitol, dexamethasone) in patients with liver failure and elevated ICP [81, 83]. These studies were graded as low quality because they variably and inconsistently assessed the effect of mannitol on ICP. Nevertheless, the results suggested some benefits of mannitol in this context. One placebo-controlled study of 30 patients with acute liver failure and elevated ICP evaluated a 3% NaCl bolus to maintain a serum sodium of 145–155 mEq/L, which increased serum sodium and significantly decreased ICP compared to placebo [82]. Retrospective analyses of patients with liver failure who received hyperosmolar therapy show conflicting results based on imaging: 23.4% NaCl reduced brain tissue volume (as measured by MRI or diffusion tensor imaging), whereas mannitol did not affect brain water or clinical status [84, 85].

The evidence identified by the panel suggests that hyperosmolar therapy can effectively treat elevated ICP or cerebral edema in the setting of fulminant liver failure and HE; however, more research is needed to determine optimal treatment strategies. The availability of a well-designed prospective clinical trial comparing HTS and mannitol in this patient population would substantially change the strength of evidence in this area. In addition, a gap in the literature exists regarding the influence of ammonia-lowering therapy on ICP, cerebral edema, or neurological outcomes.

### Hyperosmolar Therapy Safety and Infusion Considerations

- In patients receiving mannitol, does osmolarity or osmolar gap best predict the likelihood for AKI?
- In patients receiving hypertonic sodium solutions, does the serum sodium concentration best predict toxicity [AKI, unwanted acidosis] compared to the serum chloride concentration?

#### Recommendations for Assessing the Risk of Renal Injury After Mannitol Administration

1. We suggest using osmolar gap over serum osmolarity thresholds during treatment with mannitol to monitor for the risk of AKI (conditional recommendation, very low-quality evidence).

##### *Rationale*:

In making this recommendation, the panel felt that the quality of evidence was very low. While osmolar gap has not been definitively shown to predict AKI during mannitol treatment, the osmolar gap appears to correlate best with mannitol concentration and elevated mannitol concentration is best associated with toxicity. Thus, the physiological rationale for the use of osmolar gap is stronger than using an empiric osmolarity threshold.

2. There is insufficient evidence to recommend a cutoff value for osmolar gap when evaluating for the risk of acute kidney injury.

##### *Rationale*:

In making this recommendation, the panel felt that the quality of evidence was too low to make a definitive recommendation. While the panel recognizes that an osmolar gap of 20 mOsm/kg has been used in clinical practice, we were unable to identify evidence to support this threshold. Research suggests that serum mannitol concentrations are the most effective method to assess but AKI risk, but this laboratory measurement is not commonly available. We did identify one study that demonstrated an osmolar gap of 55 mOsm/kg or higher being most correlated with serum mannitol concentration.

3. Renal function measures should be monitored closely in patients receiving mannitol due to the risk of AKI with hyperosmolar therapy (good practice statement).

The panel evaluated whether osmolar gap was more predictive of AKI than osmolarity threshold in neurocritical care patients receiving mannitol (Table 1, Question 13). Three publications have addressed this specific issue, though no studies directly compare the two measures [86–88]. Because of this lack of direct comparison, we were unable to make a strong recommendation for one monitoring strategy over the other. The panel defined outcomes of interest as any related to renal dysfunction. The overall quality of evidence was very low (Table 11).

The incidence of AKI is estimated to be between 6 and 12% of patients treated with mannitol [88, 89]. Several specific risk factors have been identified in patients with mannitol-associated AKI, including heart failure, diabetes, higher severity of illness (APACHE II or NIHSS), and pre-existing renal dysfunction [88, 89]. The precise mechanism for mannitol-related AKI is not well defined, but appears to be concentration-related [90].

Very limited evidence is available to guide clinicians in choosing an osmolarity parameter when administering mannitol. Clinicians commonly use a serum osmolarity of 320 mOsm/kg or an osmolar gap of 20–55 mOsm/kg to estimate the risk of AKI with mannitol; both are indirect surrogates of serum mannitol concentration [91]. Serum mannitol concentration is possibly the best indicator of AKI risk based on animal models, however most clinical laboratories are not able to directly measure this [92]. Of the clinical measures serum mannitol concentration appears to correlated best with osmolar gap [86, 91]. In one case series of eight patients with mannitol-induced AKI, an elevated osmolar gap was present in all but two patients (mean osmolar gap 74 mOsm/kg) [86]. Early publications describing a serum osmolarity threshold of 320 mOsm/L included variable mannitol dosing regimens under different treatment paradigms that may no longer apply to current therapy (e.g., continuous infusion) [87, 93]. Recent studies have shown that an osmolarity threshold of greater than 320 mOsm/L does not affect the incidence of AKI [88]. Clinicians should monitor intravascular volume status, renal function, and some measure of serum osmolarity closely when using mannitol in patients with cerebral edema.

#### Recommendations for Assessing the Risk of Toxicity (Acute Kidney Injury or Unwanted Acidosis) After Hypertonic Sodium Solution Administration

1. We suggest that severe hypernatremia and hyperchloremia during treatment with hypertonic sodium solutions should be avoided due to the association with acute kidney injury (conditional recommendation, low-quality evidence). An upper serum sodium range of 155–160 mEq/L and a serum chloride range of 110–115 mEq/L may be reasonable to decrease the risk of acute kidney injury (conditional recommendation, very low-quality evidence).

##### *Rationale*:

In making this recommendation, the panel rated the quality of evidence as very low. The precise serum values associated with acute kidney injury varies across the literature. Clinicians should evaluate the appropriate sodium and chloride concentrations in individual patients based on renal function, acid–base balance, and the need for acute treatment for elevated ICP or cerebral edema.

2. Clinicians should routinely monitor both sodium and chloride serum concentrations to assess risk of AKI related to elevated concentrations (good practice statement).
3. Renal function should be monitored closely in patients receiving hypertonic sodium solutions due to the risk of AKI with hyperosmolar therapy (good practice statement).

##### *Rationale*:

In making these good practice statements on monitoring renal function, serum sodium, and serum chloride, the panel felt that the practice of routine monitoring for acute kidney injury was prudent given the potential risks of both HTS in this setting. Clinicians should use patient-specific factors to determine the frequency of monitoring with ranging from twice daily to every 2 h based on rapidity of change to interventions.

The panel evaluated the safety of HTS as it relates to the development of AKI and unwanted metabolic acidosis (Table 1, Question 14). Hypernatremia and hyperchloremia have both been linked to AKI and poor outcomes in a variety of critical care populations [94–97]. A small number of publications directly compared serum sodium concentrations to serum chloride concentrations in terms of the risk of AKI. We identified six studies that evaluated the relationship between use of HTS, hypernatremia, and AKI. Varying concentrations of HTS, administration techniques, and serum sodium ranges (generally 155–160 mEq/L) have been described in the literature, which complicates interpretation [98, 99]. Also included in the panel’s analysis were several studies that commented on the safety and/or side effects of hyperosmolar therapy in the neurocritical care setting but were not designed to specifically evaluate AKI. The panel defined outcomes of interest as any related to renal dysfunction or acid–base balance. The overall quality of evidence was very low (Table 12).

The only study which was designed to distinguish between serum sodium and serum chloride in predicting AKI retrospectively reviewed the use of continuous infusion 3% NaCl in a mixed neurocritical care population [100]. Sixteen percent of patients developed AKI, which was associated with a longer intensive care unit (ICU) length of stay and a greater in-hospital mortality. Five independent risk factors for AKI were identified: severe hypernatremia (serum sodium > 155 mEq/L), male gender, African-American ethnicity, history of chronic kidney disease, or having received piperacillin/tazobactam. Hyperchloremia (serum chloride > 110 mEq/L), severe hypernatremia, and hyperosmolarity were more common in the AKI group. Other studies of neurocritical care patients who received HTS have conflicting results with regard to the relationship between hypernatremia and AKI [8, 9, 22, 30, 99, 101–103]. There is considerable variability in study design, population, statistical validity, and outcome reporting that precludes forming strong recommendations.

Hyperchloremia has been implicated in the development of AKI in several observational studies [104, 105]. In one retrospective cohort study of patients with SAH, treatment with HTS was more common in the group of patients who developed AKI. Both increase in mean serum chloride level (OR 7.39 per 10 mEq/L increase) and use of HTS (OR 2.074) were found to be independently associated with AKI. In addition, five independent risk factors for AKI were identified: male gender, hypertension, diabetes mellitus, abnormal baseline creatinine, and degree of increase in mean serum chloride concentration [104]. A separate, propensity-matched retrospective cohort study which included patients with ICH found that those who received continuous infusion 3% NaCl and developed hyperchloremia (Cl > 115 mEq/L) had significantly higher rates of AKI [106]. One retrospective cohort comparison evaluated the effects of bolus dose and continuous infusion 3% NaCl on AKI [98]. Hyperchloremia and AKI were more observed to be more frequent in the continuous infusion cohort.

Three studies have described the effect of HTS on acid–base balance. Two retrospective cohort studies described the use of a sodium acetate and NaCl mix (to equal a 2 to 3% solution) which demonstrated no difference in the rate of metabolic acidosis [101, 107]. Tseng et al. reported on the use of 23.5% NaCl to achieve a goal serum sodium of 145–155 mEq/L in patients with SAH [16]. No significant changes in serum bicarbonate concentration were noted. Despite the relative lack of studies demonstrating substantial acid–base imbalances with HTS use, clinicians should be mindful of the potential for hyperchloremia to induce metabolic acidosis, which may stimulate respiratory drive.

Though the quality of the evidence is low, it suggests that severe hypernatremia and hyperchloremia from HTS are associated with AKI. In addition, the impact of HTS on acid–base balance is not well defined in studies including neurocritical care patients. Clinicians should monitor renal function, electrolytes, and acid–base balance closely when using HTS.

### Recommendations for the Optimal Administration Method of Hypertonic Sodium Solution

- In patients with cerebral edema, how does continuous infusion of hypertonic sodium solutions compare to bolus infusion of hypertonic sodium solutions in improving neurological outcomes?

1. There is insufficient evidence to support use of a continuous infusion of HTS targeting a serum sodium goal for the purpose of improving neurological outcomes.
2. Due to insufficient evidence, we cannot recommend a specific dosing strategy for HTS to improve neurological outcomes in patients with cerebral edema.
3. Clinicians should avoid hyponatremia in patients with severe neurological injury due to the risk of exacerbating cerebral edema (good practice statement).

The panel evaluated whether titrating a continuous infusion of HTS to a target serum sodium concentration compared to the use of bolus/symptom-based dosing resulted in improved neurological outcomes in patients with cerebral edema across a variety of neurocritical care conditions (Table 1, Question 15). Six publications have specifically addressed the issue of how HTS was administered to patients, but the panel only identified one that directly compared continuous infusion versus bolus administration for control of ICP that met the criteria for this PICO. The overall quality of evidence was very low (Table 13).

While the use of a continuous infusion of 3% NaCl is believed to be a common practice in neurocritical care, this strategy is not well studied. Maguigan et al. directly compared continuous versus bolus dose 3% NaCl for control of ICP elevations in a retrospective study of 162 patients with severe TBI to assess which method was the most effective [98]. No significant difference was found in time to the goal osmolarity, although more patients receiving continuous 3% NaCl reached the goal serum osmolarity than patients receiving bolus dosing. There was no difference in ICP or mortality between the two groups.

Given the lack of evidence comparing these dosing strategies, it is not clear whether continuous or bolus dosing is preferable when targeting a goal serum sodium concentration. Additionally, there is a significant gap in the literature regarding the value of targeting a specific serum sodium concentration in patients with cerebral edema, if it is in fact efficacious. While this discussion focuses on increasing sodium concentrations to treat elevated ICP and cerebral edema, clinicians must maintain awareness of the risk and benefits of sudden sodium changes, particularly in patients with chronic hyponatremia) [108].

### Non-pharmacologic Treatment of Cerebral Edema and Elevated Intracranial Pressure

- In patients with cerebral edema, how do non-pharmacological interventions compare to pharmacological interventions for reduction of cerebral edema?

#### Recommendations for the Non-pharmacological Treatment of Cerebral Edema

1. We suggest that elevating the head of the bed to 30 degrees (but no greater than 45 degrees) be used as a beneficial adjunct to reduce intracranial pressure (conditional recommendation, very low-quality evidence).

##### *Rationale*:

In making this recommendation, the panel rated the quality of available evidence as very low. However, this intervention has been used extensively in the clinical setting and the risk of elevating the head of the bed is generally very low and may be beneficial.

2. We recommend that brief episodes of hyperventilation can be used for patients with acute elevations in intracranial pressure (strong recommendation, very low-quality evidence).

##### *Rationale*:

In making this recommendation, the panel rated the quality of available evidence was very low. However, a strong recommendation was felt to be appropriate given the extensive amount of practical experience with this therapeutic strategy. Clinicians should be mindful of the limitations of acute hyperventilation related to cerebral blood flow and the extent of PaCO_2_ reduction. Clinicians should also maintain careful awareness of the duration of therapy to avoid deleterious changes in cerebral perfusion.

3. We suggest that the use of CSF diversion be considered as a beneficial adjunct to reduce intracranial pressure (conditional recommendation, very low-quality evidence).

##### *Rationale*:

In making this recommendation, the panel felt that the quality of evidence was very low. Clinicians should assess the risks and benefits of CSF diversion using patient-specific factors.

4. While non-pharmacological interventions may be effective for acute elevations in intracranial pressure, there is insufficient evidence that non-pharmacological interventions are effective for the treatment of any specific physiological changes that produce brain swelling related to cerebral edema.

The panel evaluated whether non-pharmacologic therapies such as hyperventilation, head of the bed elevation, and CSF diversion can be used to reduce ICP and cerebral edema in neurocritical care patients (Table 1, Question 16). It must be noted that these non-pharmacological interventions decrease ICP through mechanical or structural changes without actually having any impact on cerebral edema and brain swelling. Other non-pharmacologic therapies such as surgical decompression and therapeutic hypothermia were not included, as they are adequately addressed by other guidelines [3]. The overall quality of evidence was very low (Table 14).

The panel identified nine studies which assessed ICP in relation to elevation of the head of the bed [109–117]. Compared to supine positioning, ICP was consistently lower in patients at angles of 15^o^ to as high as 90 ^o^. The effect of head of bed elevation may be attributable to cardiovascular physiology and cerebral blood volume, which can at times manifest as alterations in CPP. In fact, several studies suggest that CPP is slightly reduced as the extent of head of bed elevation increases, though not to a clinically significant degree. One prospective observational study of patients with TBI suggested that ICP was reduced and CPP was stable when elevating the patients’ head of the bed from supine to 30^o^ [113]. However, further increases in the angle of head elevation above 45^o^ may increase ICP and/or reduce CPP [109, 113, 116].

The panel identified four studies evaluating the use of transient hyperventilation for reducing ICP; studies of prophylactic and/or prolonged hyperventilation were not considered for the final recommendations. Three heterogeneous studies evaluated the effect of transient hyperventilation on ICP with variable results. Oertel et al. completed 57 trials of hyperventilation in 27 patients with TBI and noted a significant reduction in ICP in 96.5% of the patients [118]. Fortune et al. performed transient hyperventilation in 16 episodes of elevated ICP occurring in 22 patients with TBI and noted a significant reduction in ICP in 88% of the episodes [119]. In contrast, Soustiel et al. found no significant change in ICP in response to transient hyperventilation [120]. Muizelaar et al. evaluated the impact of prophylactic hyperventilation on ICP and neurological outcomes in 113 patients with TBI and found that prophylactic hyperventilation (PaCO_2_ of 25 mmHg) was associated with a more stable ICP course compared to the normal ventilation group (PaCO_2_ of 35 mmHg) [121]. There was no difference in long-term outcomes, and a subgroup analysis suggested that prophylactic hyperventilation may be harmful in patients with a motor subscore of 4–5 on the GCS. While the quality of the literature for transient hyperventilation is graded as very low, clinical experience with this practice as a temporizing treatment for elevated ICP is extensive. The use of transient hyperventilation in patients with symptoms suggesting acute cerebral edema or herniation has been a standard practice in multiple clinical settings (pre-hospital, emergency department, ICU and operating room), and clinicians have seen improvement in pupil diameter, physical examination, or ICP measurements in response to this intervention. In addition, the harm associated with hyperventilation is generally related to the risk for cerebral ischemia with prolonged vasoconstriction. Given the potential life-threatening nature of elevated ICP, lack of apparent associated harm, and possible short-term benefit with transient hyperventilation, we felt that virtually all clinicians would choose hyperventilation as an adjunctive treatment strategy given the possibility that it may decrease ICP. Thus, the panel felt that a strong recommendation was warranted.

The panel identified four studies evaluating the efficacy of CSF diversion in reducing ICP [122–125]. Physiologically, it may be reasonable to assume that CSF drainage can lower ICP by decreasing the volume of the intracranial compartment by shunting CSF from the ventricles (as opposed to decreasing cerebral edema). Overall, CSF diversion appeared to be effective at reducing the ICP in two separate prospective, observational cohort studies [122, 123]. There was a significant change in ICP immediately after drainage without a measurable improvement in other indices of cerebral perfusion. In addition, Nwachuku et al. found that continuous CSF drainage results in a lower mean ICP and overall ICP burden compared to intermittent drainage [125].

## Summary

The pharmacologic treatment of cerebral edema should be guided whenever possible by the underlying pathology. The available evidence suggests hyperosmolar therapy may be helpful in reducing ICP elevations or cerebral edema in patients with SAH, TBI, AIS, ICH, and HE, although neurological outcomes do not appear to be affected. This finding is consistent with many other interventions used in the acute care of patients with neurological conditions in that treatments may affect an immediate abnormality, but outcomes are often influenced by multiple factors that may be beyond the awareness or control of the treating team (e.g., comorbidities, associated injuries, rehabilitation availability, etc.). Corticosteroids appear to be helpful in reducing cerebral edema in patients with bacterial meningitis, but not ICH. Differences in therapeutic response and safety may exist between HTS and mannitol. The use of these agents in these critical clinical situations merits close monitoring for adverse effects.

The various treatments for ICP or cerebral edema reviewed in this guideline are used in patients with critical intracranial pathologies worldwide. Despite their ubiquity, the overall quality of the literature in this area is low and there is a paucity of rigorous prospective trials. The strength of the panel’s recommendations was frequently downgraded due to the limited evidence available comparing the different treatments. The concepts of hyperosmotic therapy have been entrenched in the critical care management of patients with neurological injuries for years, yet there is limited evidence to guide clinicians on the optimal practical use of mannitol or hypertonic saline. There is a dire need for high-quality research to better inform clinicians of the best options for the individualized care of patients with cerebral edema. The goals of research activities in this area should address specific questions, adequately reflect the scope of planned patient enrollment, and select reasonable outcome measures. Basic criteria of research efforts should include a definition of how cerebral edema is being measured, the specific purpose of any intervention (e.g., resuscitation, management of ICP, effect on physiological parameters), and what outcome measures will be assessed. In addition, research efforts should consider and report on the osmolar equivalents of agents used in the management of cerebral edema, assess the effects of specific ranges of sodium levels on markers of edema, evaluate the best duration of therapy, standardize and report basic care protocols, and consider the impact of all pharmacological and non-pharmacological interventions on designated outcomes. Finally, while the goal of all interventions in the management of neurological injuries is to improve survival and minimize disability, it must be acknowledged that a single intervention to address a specific physiological variable may have limited impact on the multiple factors that contribute to both short- and long-term outcomes.

## Electronic supplementary material

Below is the link to the electronic supplementary material.

Supplementary material 1 (DOCX 24 kb)

Supplementary material 2 (DOCX 80 kb)

## Abbreviations

AIS: Acute ischemic stroke
AKI: Acute kidney injury
APACHE: Acute physiology, age, and chronic health evaluation
CE: Cerebral edema
CNS: Central nervous system
CPP: Cerebral perfusion pressure
CSF: Cerebrospinal fluid
CT: Computerized tomography
GCS: Glasgow coma score
GOS: Glasgow outcome scale
GOSE: Glasgow outcome scale extended
HE: Hepatic encephalopathy
HES: Hydroxyethyl starch
HTS: Hypertonic sodium solution (usually referring to sodium chloride 3%, 7.5%, or 23.4%, but also inclusive of solutions ranging from 1.5 to 23.5% and various other sodium salts including lactate and bicarbonate)
ICH: Intracerebral hemorrhage
ICP: Intracranial pressure
mRS: Modified Rankin scale
NaCl: Sodium chloride
NIHSS: National Institutes of Health Stroke Scale
PICO: Population, intervention, comparison, outcome
RCT: Randomized controlled trial
SAH: Subarachnoid hemorrhage
TB: Tuberculosis
TBI: Traumatic brain injury
TTH: Transtentorial herniation
