# Supplementary material for: Guidelines for the Acute Treatment of Cerebral Edema in Neurocritical Care Patients
Source: Neurocrit Care. 2020 May 15;32(3):647–66. doi: 10.1007/s12028-020-00959-7 (PMC7272487; doi:10.1007/s12028-020-00959-7)
Supplement: Supplementary file 1 — Supplementary material 1 (DOCX 24 kb) [file 12028_2020_959_MOESM1_ESM.docx]

Summary of Findings Table

| Category | Interventions of Interest | Comparators of Interest | Outcome | Quality of Evidence (QoE) Concerns^A^ | | | | QoE  Rating | Summary of findings |
| --- | --- | --- | --- | --- | --- | --- | --- | --- | --- |
|  |  |  |  | Risk of bias | Inconsistency | Indirectness | Imprecision |  |  |
| SAH | HTS 3% infusion or 23.4% bolus dose to target sodium 145-155 mEq/L | None, historical controls | ICP/CE control | Serious | Not Serious | Serious | Serious | Very low | Data on sodium target-based HTS dosing for ICP control was extremely limited & provided only indirect evidence in patients with SAH |
| SAH | Symptom-based, bolus dosing of HTS 23.4-23.5% or 7.25% / 6% HES | None, 0.9% NaCl | ICP/CE control | Serious | Not serious | Serious | Serious | Very low | Consistency of literature justified symptom-based, bolus dosing of HTS as an effective means of reducing ICP /CE in patients with SAH |
| TBI | Various forms of HTS or mannitol^B^ | None, historical controls, non-pharmacologic interventions, various forms of fluids, HTS or mannitol^C^ | ICP/CE control | Serious | Not serious | Serious | Serious | Low | Consistency of literature suggesting HTS was at least as safe & effective as mannitol in addition to the putative advantages of HTS over mannitol for fluid resuscitation & cerebral perfusion justified the suggestion to use HTS over mannitol in patients with TBI |
| TBI | Various forms of HTS or mannitol^D^ | None, historical controls, various forms of fluids, HTS or mannitol^E^ | Neurologic outcome | Serious | Not serious | Serious | Serious | Low | Use of HTS or mannitol has not been demonstrated to improve neurological outcomes in patients with TBI |
| AIS | Various forms of HTS ^F^ | None; historical controls; various forms of mannitol^G^ | ICP/CE control | Serious | Serious | Serious | Serious | Very low | Overall literature in patients with AIS was not compelling to recommend one agent over the other for treatment of ICP/CE; Patient-specific factors may be employed to aid clinicians in selecting the appropriate initial agent |
| AIS | Various forms of HTS^H^ | None; historical controls; various forms methods of mannitol ^G,I^ | Neurologic outcome | Serious | Serious | Serious | Serious | Very low | Use of HTS or mannitol has not been demonstrated to improve neurological outcomes in patients with AIS; the lack of benefit & the potential association with worse neurologic outcomes justified avoiding prophylactic mannitol in patients with AIS |
| ICH | HTS 23.4% symptom-based, bolus dosing or 3% continuous infusion with target sodium of 145-155 mEq/L | None; historical controls, symptom-based, bolus dosing of mannitol 20% | ICP/CE control | Serious | Not serious | Serious | Serious | Very Low | Consistency of literature suggesting HTS was at least as safe & effective as mannitol in addition to the putative advantages of HTS over mannitol for fluid resuscitation & cerebral perfusion justified the suggestion to use HTS over mannitol in patients with ICH |
| ICH | Dexamethasone | Placebo, none, standard of care | Neurologic outcome | Serious | Not serious | Not serious | Not serious | Moderate | Corticosteroid use associated with increased rates of complications & no improvement in neurologic outcomes |
| Bacterial meningitis | Dexamethasone^J^ | Placebo, none, historical controls | Neurologic outcome | Serious | Not serious | Not serious | Not serious | Moderate | Dexamethasone use associated with reduced neurologic sequelae (primarily hearing loss) when initiated prior to or with the first dose of antibiotics in patients with community-acquired meningitis |
| TB meningitis | Corticosteroids^K^ | Placebo, none, historical controls | Neurologic outcome | Serious | Serious | Not serious | Not serious | Moderate | Corticosteroid use associated with reduced mortality in patients with TB meningitis; one specific corticosteroid or dose in patients with TB meningitis cannot be recommended due to the inconsistency of agents & doses evaluated in the literature |
| CNS infections (bacterial & TB meningitis) | HTS & rescue mannitol^L^ | None | ICP/CE control | Serious | Not serious | Serious | Serious | Very low | Insufficient evidence to determine whether HTS or mannitol is more effective to reduce ICP or cerebral edema in patients with community-acquired bacterial meningitis; no evidence available related to TB meningitis |
| Hepatic encephalopathy | Varying forms of HTS & mannitol^M^ | None, standard of care | ICP/CE control | Serious | Not serious | Serious | Serious | Very low | Literature in patients with hepatic encephalopathy was not compelling to recommend one method of administration of HTS over the other for initial management of elevated ICP or cerebral edema; patient-specific factors may be employed to aid clinicians in selecting the appropriate initial agent |
| Hepatic encephalopathy | Varying forms of HTS & mannitol^M^ | None, standard of care | Neurologic outcome | Serious | Not serious | Serious | Serious | Very low | Insufficient evidence to determine if either hyperosmolar therapy or ammonia-lowering therapy improves neurological outcomes in patients with hepatic encephalopathy |
| Safety | Symptom-based & scheduled, bolus dosing every 6 hr of mannitol 20% | None | AKI | Serious | Not serious | Serious | Serious | Very low | Osmolar gap appears to correlate best with mannitol concentration & elevated mannitol concentration is best associated with toxicity; osmolar gap of 20mOsm/kg has been used in clinical trials as a safety threshold, but the osmolar gap of 55mOsm/kg appears to correlate best with mannitol concentrations that are associated with the development of AKI |
| Safety | Various forms of HTS^N^ | None, historical controls, various forms of fluids, HTS, or mannitol^O^ | AKI | Serious | Not serious | Serious | Serious | Very low | Precise serum values associated with acute kidney injury varies across the literature; clinicians should evaluate the appropriate sodium & chloride concentrations in individual patients based on renal function, acid-base balance, & the need for acute treatment for ICP/CE |
| Administration Method | 3% HTS continuous infusion | Symptom-based, bolus 3% HTS | Neurologic outcome | Serious | Not serious | Serious | Serious | Very low | Insufficient evidence to support use of a continuous infusion of HTS targeting a serum sodium goal for the purpose of improving neurological outcomes |
| Non-pharmacologic therapy | Head of bed elevation from 0 to 90 degrees | Standard of care | ICP/CE control | Serious | Not serious | Serious | Serious | Very low | Elevating the head of bed has been used extensively in the clinical setting & the risk of this intervention is generally very low & may be beneficial |
| Non-pharmacologic therapy | Hyperventilation | Standard of care, normal ventilation | ICP/CE control | Serious | Not serious | Serious | Serious | Very low | Extensive amount of practical experience with hyperventilation; clinicians should be mindful of the limitations of acute hyperventilation related to cerebral blood flow & the extent of PaCO_2_ reduction |
| Non-pharmacologic therapy | Cerebrospinal fluid drainage | Standard of care, historical controls | ICP/CE control | Serious | Not serious | Serious | Serious | Very low | Clinicians should assess the risks & benefits of CSF diversion using patient-specific factors |

^A^No reasons for upgrade were identified for any studied evaluated;

^B^ Symptom-based, bolus dosing of the following: 1.6% NaCl, 3% NaCl, 7.2% NaCl, 7.45% NaCl, 7.5% NaCl, 10% NaCl, 15% NaCl, 20% NaCl, 23.4% NaCl, Na Lactate, 8.4% NaHCO_3_, 7.5% NaCl / 6% HES, 7.5% NaCl / 6% Dextran-70, Mannitol 15 & 20%; Continuous infusions of the following: 7.2% NaCl / HES 200/0.5 without a sodium target, 2-3% NaCl / Acetate with target sodium 145-155 mEq/L; 20% NaCl with target sodium determined by treating physician, 3% NaCl without a sodium target, 3% NaCl with target sodium 145-155 mEq/L;

^C^ Symptom-based, bolus dosing of the following: 3% NaCl, 5% NaCl, 7.5% NaCl, 23.4% NaCl, mannitol 20%; scheduled mannitol 20% every 2 hr; bolus dosing of lactated ringers with 0.45% NaCl continuous infusion; mannitol 15% continuous infusion;

^D^ Symptom-based, bolus dosing of the following: 1.6% NaCl, 3% NaCl, 7.2% NaCl, 7.5% NaCl, 23.4% NaCl; Na lactate; 8.4% NaHCO_3_; 7.5% NaCl / 6% HES, mannitol 20%; Continuous infusions of the following: 2-3% NaCl / Acetate with target sodium 145-155 mEq/L; 7.2% NaCl / HES 200/0.5 without a sodium target; 20% NaCl with target sodium determined by treating physician; 3% NaCl without a sodium target; Prehospital bolus dosing of the following: 7.5% NaCl, 7.5% NaCl/ 6% Dextran-70, mannitol 20%

^E^ Symptom-based, bolus dosing of mannitol 20%; Continuous infusion of mannitol 15%; scheduled mannitol 20% every 2 hr; 0.9% NaCl continuous infusion; lactated ringers bolus dosing with 0.45% NaCl continuous infusion, Prehospital bolus dosing of 0.9% NaCl & lactated ringers;

^F^ Symptom-based, dosing of the following: 14.1% NaCl, 23.4% NaCl, 10% NaCl as rescue therapy in patients unresponsive to mannitol, 7.5% NaCl / 6% HES; Continuous infusion of the following: 3% NaCl/Acetate or 3% NaCl to target sodium of 145-155 mEq/L; 7.2% NaCl/HES without a sodium target;

^G^ Symptom-based, bolus dosing of mannitol 20%; Continuous infusion of mannitol 15%

^H^ Symptom-based, bolus dosing of the following: 5.1-7.6% NaCl, 23.4% NaCl; Continuous infusion of the following: 7.2% NaCl/HES continuous infusion without a sodium target, 3% NaCl continuous infusion to a goal sodium of 145-155 mEq/L

^I^ Symptom-based dosing of mannitol 18% or scheduled bolus dosing every 6 hr of mannitol 20%;

^J^ A small number of studies evaluated hydrocortisone, prednisone, or prednisolone & demonstrated no improvements in the outcomes of interest;

^K^ Corticosteroids studied included the following: dexamethasone, hydrocortisone, prednisone, or methylprednisolone;

^L^ Formation of HTS and mannitol not listed;

^M^ Symptom-based, bolus dosing of the following: 23.5% NaCl, mannitol 20%; Continuous infusion of the following: 30% NaCl infusion with a target sodium 145-155 mEq/L, mannitol 20%;

^N^ Symptom-based, bolus dosing of the following: 3% NaCl, 3% NaCl / Acetate, 7.2% NaCl, 7.5% NaCl, 23.4% NaCl; Continuous infusion of the following without a goal sodium: 3% NaCl / Acetate, 2-3% NaCl; Continuous infusion of the following with a goal sodium of 145-155 mEq/L: 2-3% NaCl / Acetate, 3% NaCl; Bolus dosing of 23.5% NaCl with a goal sodium of 145-155 mEq/L;

^O^ Symptom-based, bolus dosing of 3% NaCl, 7.5% NaCl, mannitol 20%; 0.9% NaCl bolus or infusion
