# Supplementary material for: Guidelines for the Acute Treatment of Cerebral Edema in Neurocritical Care Patients
Source: Neurocrit Care. 2020 May 15;32(3):647–66. doi: 10.1007/s12028-020-00959-7 (PMC7272487; doi:10.1007/s12028-020-00959-7)
Supplement: Supplementary file 2 — Supplementary material 2 (DOCX 80 kb) [file 12028_2020_959_MOESM2_ESM.docx]

Risk of Bias Table 2. Studies evaluating use of hypertonic sodium solutions in patients with SAH

| Reference | Patient number | Study design | Intervention | Comparator | End points of interest | Findings | Risk of bias (high/low/unclear) | Comments on risk of bias |
| --- | --- | --- | --- | --- | --- | --- | --- | --- |
| Tseng 2003 (8) | 10 SAH | P | 23.5% NaCl  2 ml/kg | None | ICP, CPP, CBF | ICP decreased by 74.7%, CPP increased by 26.8%, & xenon CT demonstrated increased CBF of 22.9% following 23.5% NaCl bolus | High | Lack of comparator, imprecision, Indirectness |
| Bentsen 2004 (13) | 7 SAH | P | 7.2% NaCl / 6% HES 2 ml/kg | None | ICP, CPP | Mean maximum ICP decrease from baseline was 58% with mean percent peak increase in CPP of 26% | High | Lack of comparator, imprecision, Indirectness |
| Al-Rawi 2005 (15) | 14 SAH | P | 23.5% NaCl  2 ml/kg | None | ICP, CPP, CBF | Reduced ICP with increased CPP & flow velocity 30 min after 23.5% NaCl dose (p<0.05); at 60 min, only ICP & flow velocity changes were significant | High | Potentially missing outcome data, Lack of comparator, imprecision, Indirectness |
| Bentsen 2006 (11) | 22 SAH | RCT | 7.2% NaCl / 6% HES  2 ml/kg (n=11) | 0.9% NaCl  2 ml/kg (n=11) | ICP | Mean reduction in ICP was 3.3 + 2.6 mmHg in the intervention group vs. 0.3 + 1.3 mmHg in comparator group | Low | Imprecision |
| Tseng 2007 (16) | 35 SAH | P | 23.5% NaCl  2 ml/kg with goal Na 145-155 mEq/L | None | ICP, CPP, CBF, mRS | Maximum CPP increase of 21.2% followed by a 93.1% maximum decrease in ICP; 31.4% mortality rate with 60% of patients having a mRS 4-6 at discharge; dose-dependent effect of CBF increments on favorable outcome was seen on xenon CT scans | High | Lack of comparator, imprecision, Indirectness |
| Bentsen 2008 (12) | 20 SAH | R | 7.2% NaCl / 6% HES 0.9-2 ml/kg | None | ICP | Mean ICP wave amplitude decreased 3.4 mmHg from baseline | High | Lack of comparator, imprecision, Indirectness |
| Koenig 2008 (10) | 68 NCC (16 SAH) | R | 23.4% NaCl 30-60 mL | None | Clinical reversal of TTH, ICP, survival, mRS | TTH reversal observed in 75% of cases & was predicted by a >5 mEq/L rise in serum Na (OR 12.0, 95% CI 1.6-90.5) or absolute serum Na >145 mEq/L (OR 26.7, 95% CI 3.6-200.0); ICP decreased from 23.3 + 16.2 to 13.8 + 10.3 mmHg; 32.4% of patients survived to hospital discharge with 77% of survivors having a discharge mRS 4-5 | High | Lack of comparator, imprecision, indirectness |
| Al-Rawi 2010 (14) | 44 SAH | P | 23.5% NaCl 2 ml/kg | None | ICP, CPP, CBF, mortality, mRS | Significant increase in CPP & flow velocity seen with decrease in ICP (p<0.05); 12-month outcomes were 33% mortality with 64% of patients with mRS>4 | High | Lack of comparator, imprecision, Indirectness |
| Hauer 2011 (9) | 215 NCC (38 SAH) | R | 3% NaCl infusion with goal Na 145-155 mEq/L  (n=100, 16 SAH) | Historical cohort not receiving 3% NaCl (n=115, 16 SAH) | ICP, mortality | Fewer episodes of critically elevated ICP (17 vs 28, p=0.299) in SAH patients in intervention group; in-hospital mortality was not significantly improved in SAH patients | High | Imprecision, Indirectness |

Risk of Bias Table 3. Studies evaluating hypertonic sodium solutions & mannitol in TBI

| Reference | Patient number | Study design | Intervention | Comparator | End points of interest | Findings | Risk of bias (high/low/unclear) | Comments on risk of bias |
| --- | --- | --- | --- | --- | --- | --- | --- | --- |
| Levin 1979 (126) | 200 NCC (42 TBI) | R | 30% urea IV,  20% mannitol, or furosemide IV | 30% urea PO, 10% glycerol PO, thiopental IV | ICP, mortality, functional outcome | All ICP measured with an epidural monitor, with 90 patients exhibiting high ICP; equiosmolar amounts of IV urea and mannitol had similar effects on ICP; 21% of TBI patients died with 14 survivors returning to baseline functional status and 6 having moderate to severe disability | High | Imprecision, Indirectness |
| Schwartz  1984 (127) | 52 TBI | RCT | 20% mannitol 1g/kg bolus | Pentobarbital 10/kg IV bolus with 0.5-3 mg/kg/hr continuous infusion | ICP, mortality | Mannitol was more effective than pentobarbital for control of high ICP in patients with & without intracranial hematomas; in patients without intracranial hematomas, 3-month mortality was lower in patients treated with mannitol (41%) as compared with pentobarbital (77%); no difference in mortality in  patients with intracranial hematomas requiring surgical evacuation; all patients also received DEX 10 mg IV followed by 4 mg every 6 hr | High | Imprecision, Indirectness |
| Smith 1986 (128) | 80 TBI | RCT | Mannitol 20% 0.75g/kg bolus if ICP>25 mmHg | Mannitol 20% 0.75 g/kg bolus, then 0.25 g/kg every 2 hr scheduled irrespective of ICP readings | Mortality, GOS | No difference in mortality or GOS between the 2 treatments; patients in comparator group also able to receive mannitol 0.75g/kg if needed for high ICP | High | Imprecision, Indirectness |
| Midgely 1993 (129) | 9 TBI | P | Mannitol 0.5 g/kg IV bolus | Thiopentone 4 mg/kg | ICP, CPP, SJO_2_ | All patients received treatment with both agents, with success defined as CPP increased above 60 mmHg and ICP reduced <20 mmHg; thiopentone given first in 6 patients; mannitol was successful in 7 patients and thiopentone in 4 patients; SJO2 increased after all mannitol doses, but fell after the majority of thiopentone treatments | High | Imprecision, Indirectness |
| Smedema 1993 (130) | 14 NCC  (7 TBI) |  | Mannitol 20% 0.5 g/kg bolus | Glycerol 10% 0.5g/kg IV bolus OR glycerol 85% oral with equal volume 0.9% NaCl | ICP | 102 individual treatments analyzed; measurement of serum osmolarity and drug concentration during therapy showed a maximum rise in osmolarity for oral glycerol; oral glycerol had the longest of action with significantly larger maximum ICP reduction with both oral and IV glycerol | High | Imprecision, Indirectness |
| Fortune 1995 (119) | 22 TBI | P | HPV, mannitol 25 g IV bolus, or CSF drainage at discretion of treating physician | None | ICP, CBF | 196 elevated ICP episodes occurred with CSF drainage used in 67%, mannitol in 25%, & HPV in 8%; after mannitol, ICP fell in 90% of observations by 7.4 mmHg & resulted in the most improvement in CBF between the 3 strategies | High | Lack of comparator, Imprecision, Indirectness |
| Sayre 1996 (34) | 41 TBI | RCT | Prehospital 20% mannitol 1 g/kg (1098 mOsm/L) (n=20) | Prehospital 0.9% NaCl 5 ml/kg (308 mOsm/L) (n=21) | SBP, mortality | No difference in rates of hypotension between (SBP<90 mmHg) group; no significant difference in mortality between mannitol & NaCl groups (25% vs 15%, p = 0.38); study noted by authors to not be adequately powered to detect mortality difference | High | Imprecision, Indirectness^B^ |
| Biestro 1997 (131) | 16 TBI | RCT | 15% Mannitol 100 mL bolus (860 mOsm/L)  (n=8) | 10% Glycerol in 0.45% NaCl bolus (1300 mOsm/L)  (n=8) | ICP, CPP | Both agents led to a clinically & statistically significant decrease in ICP & increase in CPP at 1 & 2 hr post infusion; there were no significant differences in mean change in ICP or CPP at 1 or 2 hr or mean value per day | High | Imprecision, Indirectness |
| Hartl 1997 (132) | 6 TBI | P | 7.5% NaCl / 6% HES 250mL in repeated boluses  (n=32 doses) | None | ICP, CPP, GOS, mortality | 7.5% NaCl / 6% HES significantly reduced ICP (45+15 to 25+14 mmHg) & increased CPP (52+18 to 72+16 mmHg) at 30 min in patients with elevated ICP refractory to mannitol & other therapies; repeated administration was effective; 4 patients had a good 6-month outcome (GOS 5) & one patient died | High | Lack of comparator, Imprecision, Indirectness |
| Hartl 1997 (133) | 11 TBI | R | 20% mannitol 125mL (n=30 doses) | None | ICP, PbtO_2_, CPP | Mannitol significantly reduced ICP (23+1 to 16+2 mmHg at 60 min) & increased CPP (68+2 to 80+3 mmHg at 120 min) when initial ICP > 20 mmHg, but did not impact outcomes when initial ICP < 20 mmHg | High | Lack of comparator, Imprecision, Indirectness |
| Qureshi 1998 (37) | 27 NCC (8 TBI) | R | 3% NaCl/ NaAcetate infusion with goal Na 145-155 mEq/L | None | ICP, lateral displacement mass effect on CT scan | Mean GCS improved at termination of infusion compared with start of infusion; mean ICP reduced from 14.2+4.2 to 7.3+1.8 mmHg, but was not sustained; lateral displacement of brain was reduced in TBI patients within 72 hr | High | Lack of comparator, Imprecision, Indirectness |
| Shackford  1998 (134) | 34 TBI | RCT | 1.6% NaCl resuscitation & 0.9% NaCl continuous infusion  (n=18) | LR resuscitation & 0.45% NaCl continuous infusion  (n=16) | ICP, GOS | No difference in mean ICP between groups over 5 days; 1.6% NaCl patients required significantly more interventions for elevated ICP; no significant difference in mean discharge GOS | High | Imprecision, Indirectness |
| Schatzmann  1998  (135) | 6 NCC  (5 TBI) | P | 10% NaCl 100mL bolus  (n= 42 doses) | None | ICP | 10% NaCl reduced ICP by 43% from baseline values; maximum ICP reduction occurred at 24+15 min & lasted 101+50 min | High | Lack of comparator, Imprecision, Indirectness |
| Qureshi  1998 (136) | 82 TBI | R | 2% or 3% NaCl/Na Acetate infusion with goal Na 145-155 mEq/L (n=36) | 0.9% NaCl  (n=46) | Mortality, GOS | Infusion of 2-3% NaCl / Na Acetate was associated with both higher in-hospital mortality (OR 3.1, 95% CI 1.1 - 10.2) & higher discharge GOS | High | Lack of comparator, Imprecision, Indirectness |
| Horn  1999 (137) | 10 NCC (6 TBI) | P | 7.5% NaCl  2 ml/kg | None | ICP, mortality, GOS | 7.5% NaCl resulted in mean ICP decrease of 14.5 mmHg; maximum ICP lowering occurred after a mean of 98 min; 70% mortality, with one TBI survivor having a favorable outcome | High | Lack of comparator, Imprecision, Indirectness |
| Munar 2000 (138) | 14 TBI | P | 7.2% NaCl 1.5 mL/kg bolus | None | ICP, GOS | 7.2% NaCl reduced ICP by 30% without significant changes in estimated CBF; poor GOS outcome seen in 43% of patients | High | Lack of comparator, Imprecision, Indirectness |
| Vialet  2003 (19) | 20 TBI | RCT | 7.5% NaCl 2mL/kg  (4.8 mOsm/kg)  (n=10) | 20% mannitol 2mL/kg  (2.3 mOsm/kg)  (n=10) | ICP, GOS, mortality | Mean number of high ICP episodes per day (6.9 + 5.6 vs 13.3 + 14.6) & the daily duration of high ICP (67 + 85 vs 131 + 123 min) were significantly lower in 7.5% NaCl group, along with lower rate of treatment failure; 90-day mortality & GOS did not differ between groups | High | Imprecision, Indirectness |
| Cooper  2004 (32) | 229 TBI | RCT | Prehospital  7.5% NaCl 250 mL (n=114) | Prehospital  LR 250 mL  (n=115) | Mortality, GOSE | No difference in 6 months in survival rates & favorable outcomes (GOSE 5-8) | Low | Indirectness |
| Soustiel 2006 (120) | 36 TBI | P | HPV  combined with mannitol 20% 0.5 g/kg | Baseline data before intervention | CBF, ICP, CPP | Values measured at baseline, after HPV, & after mannitol 20%; HPV defined as reducing PaCO2 from 36 + 4 to 32 + 4mmHg, which did not produce a significant ICP change; CPP increased by 5.6% with a statistically significant CBF decrease; mannitol resulted in non-significant change in ICP, 5 mmHg increase in CPP, & also increased CBF | High | Imprecision, Indirectness |
| Battison  2005 (20) | 9 NCC  (6 TBI) | RCT^A^ | 7.5% NaCl / 6% dextran-70 100 mL (n=18 treatments) | 20% Mannitol 200 mL (n=18 treatments) | ICP | All patients received 2 treatments of each agent in equiosmolar doses & in a randomized order; both treatments led to a significant reduction in minimum ICP; intervention group resulted in a greater decrease in ICP (median difference 5 mmHg) & a longer duration of effect (median difference 46 min) vs mannitol | High | Imprecision, Indirectness |
| Harutjunyan  2005 (21) | 32 NCC  (10 TBI) | RCT | 7.2% NaCl/HES 200/0.5 continuous infusion  (n=17) | 15% Mannitol continuous infusion  (n=15) | ICP,  mortality | Both infusions (non-equiosmolar) continued until ICP < 15 mmHg; both treatments reduced ICP to goal, but 7.5% NaCl reduced it to a greater degree, faster, & for a longer time; mean effective dose to achieve goal ICP was 1.4 ml/kg for 7.2% NaCl/HES 200/0.5 & 1.8 ml/kg for mannitol; 41.2% mortality in NaCl/HES group compared to 60% with mannitol (p>0.05) | High | Imprecision, Indirectness^B^ |
| Ware  2005 (22) | 13 TBI | R | Mannitol bolus in varying doses (mean 0.86 g/kg)  (n=19 treatments) | 23.4% NaCl 30mL  (n=22 treatments) | ICP | All subjects treated with mannitol then 23.4% NaCl for refractory ICP; both (non-equiosmolar) treatments led to a decrease in ICP with no significant difference between agents; 23.4% NaCl resulted in a longer duration of decreased ICP vs mannitol (96 min vs 59 min; p=0.02) | High | Imprecision |
| Huang 2005 (139) | 18 TBI | P | 3% NaCl 300ml bolus (1026 mOsm/L) in place of mannitol (n=35 doses) | None | ICP, CPP, MAP, CBF | 3% NaCl replaced mannitol once a day when needed; ICP reduced (30 to 23mmHg) & CPP increased (78 to 87mmHg) at 60min; no changes in MAP with increase in estimated CBF | High | Lack of comparator, Imprecision, Indirectness |
| Lescot 2006 (140) | 14 TBI | P | 20% NaCl 40ml bolus | None | ICP, CPP, contusion volume | 20% NaCl bolus resulted in an immediate ICP decrease of 6+3 mmHg (p < 0.001) without significant changes in MAP or CPP; volume of non-contused brain decreased by 13+8 mL, while contused brain increased by 5+5 mL at 2 min | High | Lack of comparator, Imprecision, Indirectness |
| Chatterjee  2007 (141) | 25 TBI | RCT | 7.5% NaCl 2mL/kg bolus (4.8 mOsm/kg)  (n=14) | 20% Mannitol 2mL/kg bolus (2.3 mOsm/kg)  (n=11) | ICP, CPP, mortality | Mean number (7.1+2.9 vs.  14.6+3.4) & duration (62.6+28.1 vs. 93.4+37.2 min) of high ICP episodes was significantly lower in the 7.5% NaCl group while treatment failure was significantly higher in the mannitol group; no difference in mortality or neurological outcome between groups | High | Imprecision^B^ |
| Francony  2008 (23) | 20 NCC  (17 TBI) | RCT | 7.45% NaCl 100 mL  (n=10) | Mannitol 20% 231 mL  (n=10) | ICP | Equiosmolar doses (255 mOsm) of each agent equally & durably reduced ICP at 60 & 120 min; in 7.45% NaCl group, ICP was significantly reduced by 10 + 5 mmHg at 60 minutes & by 6 + 3mmHg at 120 min compared to 14 + 8 mmHg at 60 min & by 10 + 4 mmHg at 120 min in mannitol group | High | Imprecision, Indirectness |
| Koenig  2008 (10) | 68 NCC (6 TBI) | R | 23.4% NaCl 30-60 mL | None | Clinical reversal of TTH, ICP, survival, mRS | TTH reversal observed in 75% of cases & was predicted by a >5 mEq/L rise in serum Na (OR 12.0, 95% CI 1.6-90.5) or absolute serum Na >145 mEq/L (OR 26.7, 95% CI 3.6-200.0); ICP decreased from 23.3 + 16.2 to 13.8 + 10.3 mmHg; 32.4% of patients survived to hospital discharge with 77% of survivors having a discharge mRS 4-5 | High | Lack of comparator, Imprecision, Indirectness |
| Ichai  2009 (26) | 34 TBI | P^C^ | Na lactate 1.5 ml/kg (1100 mOsm/L) (n=17) | 20% mannitol  1.5 mL/kg (1160 mOsm/L) (n=17) | ICP, GOS | Na lactate solution had a significantly greater decrease (7 vs 4 mmHg; p=0.02) & more prolonged reduction in ICP after 4 hours (-5.9 mmHg vs -3.2 mmHg; p=0.009); there was a trend toward better GOS scores at 1 year in those receiving Na lactate | High | Imprecision |
| Oddo  2009 (102) | 12 TBI | R^C^ | 7.5% NaCl 250 mL  (641 mOsmol; n=14 doses) | Mannitol 25% 0.75g/kg  (412 mOsmol;  n=28 doses) | ICP | All subjects treated with mannitol then 7.5% NaCl for refractory ICP; both (non-equiosmolar) treatments resulted in a significant decrease in ICP at 60 & 120 min; 7.5% NaCl was associated with a greater decrease in ICP at 120 minutes (12 mmHg) as compared with mannitol (5 mmHg) | High | Imprecision |
| Kerwin  2009 (103) | 22 TBI | R^C^ | 23.4% NaCl 30 ml (n=108 doses) | Mannitol 20% 15 - 75g (n=102 doses) | ICP | Only difference in ICP reduction between agents occurred when ICP was > 30 mmHg & demonstrated a significantly greater reduction with 23.4% NaCl (12.6 mmHg vs 8 mmHg; p=0.01) | High | Imprecision |
| Rockswold  2009 (17) | 25 TBI | P | 23.4% NaCl 30mL bolus | None | ICP, GOS, CPP, PbtO_2_, mortality | ICP decreased by mean of 8.3 mmHg (p<0.0001); ICP reduction greater the higher the initial ICP; CPP increased by 6 mmHg & PbtO_2_ values increased by 3.1 mmHg ; 6-month mortality rate of 28% with favorable 12-month GOS outcomes in 56% | High | Lack of comparator, Imprecision |
| Bulger  2010 (33) | 1282 TBI | RCT | Prehospital  7.5% NaCl / 6% Dextran  250 mL  (n = 359)  OR Prehospital  7.5% NaCl 250 mL  (n = 341) | Prehospital  0.9% NaCl 250 mL  (n=582) | Mortality, GOSE, DRS | No significant difference in mortality at hospital discharge or 28 days; no significant differences in distribution of 6-month GOSE scores or DRS at 1-month & discharge between treatment groups | Low | Indirectness |
| Morrison  2011(142) | 107 TBI | RCT | Prehospital  250 mL 7.5% NaCl / 6% Dextran-70  (n =50)^D^ | Prehospital  250 mL 0.9% NaCl  (n=57) | Mortality, GOSE | No difference in survival rates between treatment groups at discharge or 30-days; 4-month GOSE scores were above 4 in 100% of intervention group patients vs 72% in comparator group | High | Imprecision, Indirectness |
| Cottenceau  2011 (24) | 47 TBI | RCT | 7.5% NaCl  2 mL/kg  (n=22) | 20% Mannitol  4 ml/kg  (n=25) | ICP, GOS | Equiosmolar doses of both agents effectively & equally reduced ICP, but 7.5% NaCl resulted in a stronger & longer duration of reduction; no significant difference in GOS at 6 months between groups | High | Imprecision |
| Sakellaridis  2011 (25) | 29 TBI | RCT^A^ | 15% NaCl  0.42 ml/kg bolus^E^ | 20% Mannitol 2ml/kg  Bolus^E^ | ICP | No difference in mean decrease in ICP between agents of similar osmotic burden; mean duration of effect longer with 15% NaCl (3 hr 33 min vs 4 hr 17 min; p=0.4) | High | Imprecision^B^ |
| Roquilly  2011 (143) | 50 TBI | R | 20% NaCl continuous infusion with goal Na determined by MD | None | ICP, mortality, GOS | Continuous infusion of 20% NaCl was associated with a rapid & sustained decrease in ICP at 96 hr in patients with high ICP refractory to barbiturate; 3 patients died of intractable ICP & 10 from withdrawal of care; GOS outcomes only reported for 5 patients | High | Lack of comparator, Imprecision, Indirectness |
| Bourdeaux 2011 (29) | 11 TBI | RCT | 8.4% sodium bicarbonate 85 mL (n=10 episodes) | 5% NaCl 100 mL (n=10 episodes) | ICP, mortality | Equiosmolar doses of medications administered; both treatments reduced ICP effectively at all time points; change in mean ICP at 60 min was 12.1 + 4.1 mmHg for bicarbonate & 10.1+5.1 mmHg for NaCl, but after 150 min mean ICP was higher in the 5% NaCl group; arterial pH was raised after treatment with 8.4% sodium bicarbonate; 27.3% overall mortality | High | Imprecision, Indirectness |
| Li  2015 (28) | 169 TBI | MA^F^ | Hypertonic NaCl in varying formulations & doses^E^ | Mannitol in varying dose & formulations^E^ | ICP | Hypertonic NaCl reduced ICP more than mannitol with a mean pool difference of -1.69 mmHg [95% CI -2.95 to -0.44; p=0.008]); significant difference in ICP at 60 min (pooled difference in means = -4.04 mmHg [95% CI -6.75 to -1.32; p=0.004]) favoring hypertonic NaCl | High | Imprecision, Indirectness, Inconsistency |
| Mangat  2015 (144) | 50 TBI^G^ | R | 3% NaCl (n=24), 23.4% NaCl (n=1) | 20% mannitol  (n=25) | ICP, mortality | No difference in 2-week mortality; intervention group more effective than comparator in lowering cumulative & daily ICP burdens | High | Imprecision, Indirectness |
| Colton 2016 (145) | 98  TBI | R | “Small dose” (3% NaCl in any dose < 250 mL [n=158 doses]) OR  “Large dose” (3% NaCl in any dose > 250 mL or > 100 mL 7.5% NaCl  [n=71 doses]) | Mannitol in varying doses (25-50g)  (n=7 doses) | ICP, PTD | “Small dose” reduced PTD by 38% in first hr & 37% in second hr, reduced the time with ICP >19 mmHg by 38% & 39% after 1 & 2 hr; "large dose” reduced PTD by 40% in first hr, 63% in second hr & reduced the time with ICP > 19 mmHg by 36% & 50% after 1 & 2 hr; there were no significant ICP changes after mannitol | High | Imprecision, Indirectness |
| Berger-Pelleiter  2016 (27) | 1820  TBI | MA^H^ | Hypertonic NaCl in varying formulations & doses^E^ | Alternative solution^E^ | ICP; mortality | Hypertonic NaCl did not decrease mortality or improve ICP control (weighted mean difference -1.25 mmHg, 95% CI -4.18 to 1.68) as compared to other solutions (20% mannitol, LR, 8.4% Na bicarbonate or 0.45% or 0.9% NaCl | High | Imprecision, Indirectness |
| Jagannatha 2016 (30) | 38 TBI | RCT | 3% NaCl 2.5ml/kg  (n=18) | 20% Mannitol  2.5ml/kg  (n=20) | ICP, mortality, GOS | No difference in ICP between the two equiosmolar groups over 6 days; slope of fall in ICP in response to a bolus dose at a given baseline ICP value was higher with 3% NaCl compared to mannitol (p=0.0001); in-hospital mortality tended to be lower in the 3% NaCl group while 6-month mortality was not different between groups; dichotomized 6-month GOS scores were comparable between groups | High | Imprecision |
| Tan  2016 (146) | 231 TBI | R | 3% NaCl continuous infusion  (n=124) | No 3% NaCl continuous infusion  (n=107) | ICP, mortality | 3% NaCl infusion was associated with significant decrease in ICP over 14 days; no difference in hospital mortality observed | High | Imprecision, Indirectness |
| Asehnoune  2017 (31) | 545 TBI | R | 20% NaCl continuous infusion with goal Na determined by MD  (n=143) | Standard care without continuous infusion 20% NaCl (n=402) | Mortality, GOS | 90-day survival rates favored the continuous infusion 20% NaCl group in propensity matched analysis (HR 1.74 [95% CI 1.36–2.23]; no difference in favorable outcome (GOS 4-5) between groups | High | Indirectness |
| Maguigan 2017 (98) | 162 TBI | R | 3% NaCl continuous infusion (n=132) | 3% NaCl bolus dosing (n=30) | ICP, CPP, mortality | No differences in CPP, ICP or mortality observed between groups | High | Imprecision |
| Cheng  2018 (147) | 60 TBI | R | 3% NaCl  (n=30) | Mannitol 20% (n=30) | ICP, mortality | All patients treated underwent DC & received equiosmolar doses of medications; rate of ICP decrease was higher with 3% NaCl bolus than with mannitol; no significant difference in 2-week mortality | High | Imprecision, Indirectness |
| Dunham  2018 (148) | 112 TBI | R | 3% NaCl by any administration method or dose over the first 5 days  (n=112) | None | Mortality, ability to follow commands | Overall mortality was 13.4%; lower mortality found in patients who were surgically decompressed & received >8 mEq/kg of Na; differences in ability to follow commands at 3 months were observed in multiple subgroups based upon total Na mEq received | High | Lack of comparator, Imprecision, Indirectness |
| Patil 2019 (149) | 120 TBI | RCT | 3% NaCl (1027 mOsm/L)  (n= 40) | 20% mannitol (1100 mOsm/L)  (n= 40)  OR 10% mannitol & 10% glycerol  (1049 mOsm/L)  (n= 40) | ICP, GCS, mortality | All three interventions decreased ICP below 15 mmHg (p<0.0001); maximum change in ICP occurred after a bolus of 3% NaCl followed by the mannitol / glycerol combination, then 20% mannitol; mean dose required to reduce ICP below 15 mmHg was 1.4 ml/kg for 3% NaCl, 1.7 ml/kg for mannitol/glycerol combination, & 2 ml/kg for 20% mannitol; 3% NaCl also reduced ICP faster than other agents (16 min vs 19 min vs 23 min); maximum change in GCS occurred after 3% NaCl | Low | Imprecision |
| Mangat  2019 (150) | 50 TBI^G^ | R | 3% NaCl (n=24), 23.4% NaCl (n=1) | 20% mannitol  (n=25) | Burden of combined ICP_high_ & CPP_low_ derangement | Bolus 3% NaCl is associated with a decreased combined burden of ICP_high_+CPP_low_ compared to mannitol | High | Imprecision, Indirectness |
| ^A^Cross-over study design; ^B^ Study downgraded by two levels due to very serious concerns of identified area; ^C^Rescue therapy with either agent was allowed by crossover when ICP not controlled; ^D^ Exact dosing of study medication not provided in publication, but was provided by the corresponding author of the study; ^E^ Number of patients in each study group unable to be discerned study design; ^F^ Meta-analysis included the following studies: Cottenceau 2011, Sakellaridis 2011, Oddo 2009, Francony 2008, Harutjunyan 2005, Battison 2005, Vialet 2003; ^G^ Study included two analysis with 50 patients in one (matched 1:1) & 72 patients in another (matched 1:2); ^H^ Meta-analysis included the following studies: Shackford 1998, Vialet 2003, Cooper 2004, Francony 2008, Ichai 2009, Bulger 2010, Bourdeau 2011, Cottenceau 2011, Morrison 2011, Sakellaridis 2011, Scalfani 2012  The following studies (with reason) were excluded from our assessment: Gu 2018 (meta-analysis that primarily included studies only published in Chinese that were unable to be independently verified by reviewers); Ko 2012 (unable to verify that 5 patients included received osmotherapy due to study design); Kamel 2011, Rickard 2015, Wang 2015, Burgess 2016 (meta-analysis included mixed populations of neurologic injuries without results specific to TBI reported) | | | | | | | | |

Risk of Bias Table 4. Studies evaluating use of hypertonic sodium solutions & mannitol in patients with AIS

| Reference | Patient Number | Study Design | Intervention | Comparator | End points of interest | Findings | Risk of bias (high/low/unclear) | Comments on risk of bias |
| --- | --- | --- | --- | --- | --- | --- | --- | --- |
| Santambrogio 1978 (41) | 77 AIS | RCT | Mannitol 20% 0.8-0.9 g/kg/day for 10 days (n=36) | Standard therapy with IV fluids (n=41) | Neurologic examination | No difference in neurologic examination between groups | High | Indirectness |
| Onar  1997 (151) | 52 AIS | P | Mannitol 20% 150 mL (n=31) | No osmotherapy (n=21) | SSEP, CCT improvement, N20 latency, N20-P27 amplitude | Variable improvement in all outcomes with mannitol & positive effects seen mostly in subcortical strokes | High | Imprecision, Indirectness, Inconsistency |
| Qureshi  1998 (37) | 27 NCC (6 AIS) | R | 3% NaCl/ NaAcetate infusion with goal Na 145-155 mEq/L | None | ICP, lateral displacement, mass effect on CT | Favorable trend in ICP reduction in AIS patients in intervention group, but no association between serum Na & ICP reduction; no difference in other outcomes for AIS patients | High | Lack of comparator, Imprecision, Indirectness |
| Schwarz  1998 (36) | 9 NCC (8 AIS) | RCT | 7.5% NaCl / 6% HES  100 ml (n=16 episodes) | Mannitol 20% 200 mL (n=14 episodes) | ICP, CPP | 7.5% NaCl / 6% HES was 100% successful with faster, larger & more sustained ICP reduction compared to mannitol; improvement in CPP seen even with failure of mannitol | High | Imprecision, Indirectness |
| Manno  1999 (44) | 7 AIS | P | Single dose of mannitol 1.5g/kg^A^ | None | Discharge status, MCASS, GCS, pupillary reactivity | No worsening of outcomes, variable improvement in endpoints among patients without a consistent effect | High | Lack of comparator, Imprecision, Inconsistency |
| Videen 2001 (152) | 7 AIS | P | Single dose of mannitol 1.5g/kg^A^ | None | % change in total, infarcted & normal hemispheric volume | Decrease in total brain & non-infarcted hemispheric volume with no change in infarcted hemisphere volume between baseline & follow-up MRI | High | Lack of comparator, Imprecision, Inconsistency |
| Keller  2002 (39) | 10 AIS | P | Mannitol 20% 100 mL or 100 ml of NaCl / HES^A^ | None | ICP, CBF, SjVO_2_ | Significant decrease in ICP & CBF increase after both agents with no change in SjVO2 | High | Lack of comparator, Imprecision |
| Schwarz 2002 (40) | 8 NCC (6 AIS) | P | Mannitol 20% 200 mL with 10% NaCl 75 mL if necessary | None | ICP, CPP | 10% NaCl successful in 100% of mannitol failure cases (1 with no response & 7 with suboptimal response) with significant ICP lowering & increased CPP | High | Lack of comparator, Imprecision, Indirectness |
| Bereczki 2003 (47) | 805 NCC (457 AIS) | P | Mannitol (mean dose 47 + 22 g/day) for 3-10 days (n=315) ^A^ | No osmotherapy (n=142) | Mortality | Total of 666 AIS patients, but only 457 admitted within 24 hr of symptom onset & included in analysis; similar 30-day & 1-year mortality; mannitol use did not predict survival | Low^B^ | Imprecision |
| Zuliani  2004 (45) | 442 AIS | R | Mannitol 18% 60-80 mL daily for 4-7 days (n=114) | No osmotherapy (n=130) or  glycerol (n=198) | Mortality | Mannitol associated with increased risk of death at 30-days when adjusted for age & gender; this association disappeared when adjusted for other confounders | High | Imprecision, Inconsistency |
| Harutjunyan 2005 (21) | 40 NCC (8 AIS) | RCT | 7.2% NaCl/HES 200/0.5 continuous infusion  (n=17) | 15% Mannitol continuous infusion  (n=15) | ICP, CPP  mortality | Both infusions (non-equiosmolar) continued until ICP < 15 mmHg; both treatments reduced ICP to goal, but 7.5% NaCl reduced it to a greater degree, faster, & for a longer time; mean effective dose to achieve goal ICP was 1.4 ml/kg for 7.2% NaCl/HES 200/0.5 & 1.8 ml/kg for mannitol; 41.2% mortality in NaCl/HES group compared to 60% with mannitol (p>0.05) | High | Imprecision, Indirectness^C^ |
| Koenig  2008 (10) | 68 NCC (8 AIS) | R | 23.4% NaCl 30-60 mL | None | Clinical reversal of TTH, ICP, survival, mRS | TTH reversal observed in 75% of cases & was predicted by a >5 mEq/L rise in serum Na (OR 12.0, 95% CI 1.6-90.5) or absolute serum Na >145 mEq/L (OR 26.7, 95% CI 3.6-200.0); ICP decreased from 23.3 + 16.2 to 13.8 + 10.3 mmHg; 32.4% of patients survived to hospital discharge with 77% of survivors having a discharge mRS 4-5 | High | Lack of comparator, Imprecision, Indirectness |
| Diringer  2011 (35) | 9 AIS | RCT | 23.4% NaCl 0.686ml/kg (n=4) | 20% mannitol 1g/kg (n=5) | CBF, CBV, OEF, CMRO_2_ | No change in outcomes before & after for either agent in infarct, peri-infarct or ipsilateral side; variable CBF increase in contralateral side | High | Imprecision, Indirectness, Inconsistency |
| Hauer  2011 (9) | 215 NCC (57 AIS) | R | 3% NaCl infusion with goal Na 145-155 mEq/L  (n=100,  28 AIS) | Historical cohort not receiving 3% NaCl (n=115,  29 AIS) | ICP, mortality | Fewer episodes of critically elevated ICP per AIS patient; in-hospital mortality not different between AIS patients | High | Imprecision, Indirectness |
| Strbian  2013 (42) | 49 AIS | P | 5.1-7.6% NaCl (n=29) with additional mannitol (n=4) | 10% glycerol (n=16) | Mortality, mRS | Overall 43% mortality; 10% had favorable outcome (mRS 0-2) at 3 months with all in intervention group | High | Imprecision |
| Lewandowski-Belfer 2014 (38) | 55 NCC (5 AIS) | R | 23.4% NaCl 15 or 30 mL (n=239 doses) | 14.6% HTS 24 or 48 mL (n=121 doses) | ICP | Average ICP reduction of 50% in both groups with no dose dependency | High | Imprecision |
| Ong  2015 (43) | 30 AIS | R | Mannitol (2.2g/kg/day), 23.4% NaCl (173 mmol/ kg/dose) or both | None | Medical success | Overall 47% medical success (survival to discharge without need for decompression); worse odds for success with medical therapy in those with higher NIHSS; no additive benefit of dual therapy | High | Lack of comparator, Imprecision |
| Pappagiani 2018 (46) | 922 AIS | P | Mannitol 0.2 g/kg every 6 hr for 3 days then 0.1 g/kg tapered over 5 days (n=86) | No mannitol (n=836) | Mortality, mRS | Discharge dependency (mRS 2-5) & mortality were higher with mannitol; after adjustment, mannitol was independent predictor for in-hospital & 90-day mortality | Low |  |
| ^A^ Specific formulation of medication not specified; ^B^ While risk of bias & imprecision present, they were not severe enough to warrant downgrading of evidence; ^C^ Study downgraded by two levels due to very serious concerns of identified area  The following studies (with reasoning) were excluded from our assessment: Bereczki 2007 (meta-analysis that included only one study of AIS patients; excluded to avoid duplicating the findings of this single trial) | | | | | | | | |

Risk of Bias Table 5. Studies evaluating use of hypertonic sodium solutions & mannitol in patients with ICH

| Reference | Patient Number | Study Design | Intervention | Comparator | End points of interest | Findings | Risk of bias (high/low/unclear) | Comments on risk of bias |
| --- | --- | --- | --- | --- | --- | --- | --- | --- |
| Levin 1979 (126) | 200 NCC (17 ICH) | R | 30% urea IV,  20% mannitol, or furosemide IV | 30% urea PO,10% glycerol PO, thiopental IV | ICP, mortality, functional Outcome | All ICP values measured with an epidural monitor, with 90 patients exhibiting high ICP; equimolar amounts of IV urea and mannitol had similar effects on ICP; 59% of ICH patients died with 7 survivors having moderate to severe disability | High | Imprecision, Indirectness |
| Qureshi 1998 (37) | 27 NCC (8 ICH) | R | 3% NaCl/ NaAcetate infusion with goal Na 145-155 mEq/L | None | ICP, lateral displacement mass effect on CT scan | No different in ICP outcomes in non-traumatic ICH patients treated with 3% NaCl / Na Acetate infusion | High | Lack of comparator, Imprecision, Indirectness |
| Koenig 2008 (10) | 68 NCC (29 ICH) | R | 23.4% NaCl 30-60 mL | None | Clinical reversal of TTH, ICP, survival, mRS | TTH reversal observed in 75% of cases & was predicted by a >5 mEq/L rise in serum Na (OR 12.0, 95% CI 1.6-90.5) or absolute serum Na >145 mEq/L (OR 26.7, 95% CI 3.6-200.0); ICP decreased from 23.3 + 16.2 to 13.8 + 10.3 mmHg; 32.4% of patients survived to hospital discharge with 77% of survivors having a discharge mRS 4-5 | High | Lack of comparator, Imprecision, Indirectness |
| Hauer 2011 (9) | 215 NCC (120 ICH) | R | 3% NaCl infusion with goal Na 145-155 mEq/L  (n=100,  52 ICH) | Historical cohort not receiving 3% NaCl (n=115, 68 ICH) | ICP, mortality | Fewer episodes of critically elevated ICP among ICH patients (24 vs. 60) in intervention group; in-hospital mortality was significantly decreased (11.5% vs. 27.9%; p = 0.04) in those with ICH | High | Imprecision, Indirectness |
| Wagner 2011 (49) | 90 ICH | P | 3% NaCl infusion with goal Na 145-155 mEq/L (n=26) | Historical controls (n=64) | ICP, cerebral edema, mortality | ICP crisis was observed in 34.5% of intervention group patients compared to 50% in comparator group (p=0.09), with cerebral edema reduced between day 8-14 (p=0.04); intervention group in-hospital mortality was 11.5% vs 25% in comparator group (p=0.078) | High | Imprecision, Indirectness |
| The following studies (with reasoning) were excluded from our assessment: Cruz 2004 (serious concerns of illegitimacy that were raised by scientific community(153)); Helbok 2011 (study was a mixed stroke population & only included three patients with ICH); Bereczki 2007 (meta-analysis that only included studies on ICH patients that were individually excluded from our assessment); Misra 2005, Wang 2015, Shah 2018, Aminmansour 2017 (did not meet the predetermined specifications set forth by the PICO question); Sun 2018 (meta-analysis that primarily included studies only published in Chinese that were unable to be independently verified by reviewers) | | | | | | | | |

Risk of Bias Table 6. Studies evaluating use of corticosteroids in patients with ICH

| Reference | Patient Number | Study Design | Intervention | Comparator | End points of interest | Findings | Risk of bias (high/low/unclear) | Comments on risk of bias |
| --- | --- | --- | --- | --- | --- | --- | --- | --- |
| Rubinstein  1965 (58) | 25 NCC (6 ICH) | CS | DEX 4 mg IV & 4 mg IM initially, total of 52 mg over 72 hr (n=6) | None | Mortality | All ICH patients died; autopsy performed to confirm ICH diagnosis as advanced imaging not available when study completed | High | Lack of comparator, Imprecision, Indirectness |
| Tellez  1973 (55) | 40 ICH | RCT | DEX 12 mg IV initially, then IM & tapered; total of 120 mg for 10 total days (n=19) | Placebo (n=21) | Mortality, neurologic function^B^ | Similar mortality between groups; no consistent improvement in neurologic function observed in DEX-treated patients | High | Imprecision |
| Poungvarin  1987 (62) | 93 ICH | RCT | DEX 10 mg IV initially, tapered for 9 total days (n=46) | Placebo (n=47) | Mortality, adverse events | Study was terminated after third interim analysis revealed similar mortality with increased rates of infection & diabetic complications in DEX group | Low | Imprecision |
| Desai 1998 (60) | 26 ICH | RCT | DEX 4 mg IV every 6 hr for 12 days, tapered for 16 total days (n=12) | Placebo (n=14) | GOS | No differences observed between groups in GOS scores at discharge or 7-days | High | Imprecision |
| Ogun  2001 (59) | 40 NCC (27 ICH) | RCT | DEX 100 mg IV initially, followed by 16 mg every 6 hr for 48 hr (n=15 ICH) | Placebo (n=20, 12 ICH) | Mortality | High mortality observed in both groups | High | Imprecision, Indirectness |
| Feigin 2005 (61) | 462 NCC (206 ICH) | MA^A^ | Corticosteroid therapy of any type (n= 102 ICH) | Placebo or standard of care (n=104 ICH) | Mortality, functional outcome, adverse events | All ICH trials utilized DEX; no difference in mortality or poor outcome (defined as death, severe disability, or vegetative state) with possible increased risk of adverse events | Low |  |
| Sharafadinzadeh  2008 (56) | 225 ICH | RCT | DEX 10 mg IV initially, then tapered for 10 total days (n=144) | Placebo (n=81) | Mortality | Higher mortality in DEX group compared to placebo | Low | Imprecision |
| Zaganas  2011 (57) | 850 ICH | R | Patients at one center treated with a tapering regimen of DEX IV for 10-12 days (n=340) | Patients at another center not receiving DEX (n=510) | Mortality, mRS | Lower in-hospital mortality at 30 & 90 days & lower discharge mRS scores in intervention group | High | Imprecision, Indirectness |
| ^A^ Meta-analysis included studies by Desai 1998, Hooshmand 1972, Ogun 2001, Poungvarin 1987, & Tellez 1973; Hooshmand 1972 did not meet the inclusion criteria for our guideline review as both canine & human data were evaluated; ^B^ Neurological function based upon a study-specific scoring tool with patients scored on day 2, 4, 6, 8, 10 & 14 after admission into study | | | | | | | | |

Risk of Bias Table 7. Studies evaluating the use of adjunctive corticosteroid therapy in bacterial meningitis

| Reference | Patient Number | Study Design | Intervention | Comparator | End points of interest | Findings | Risk of bias (high/low/unclear) | Comments on risk of bias |
| --- | --- | --- | --- | --- | --- | --- | --- | --- |
| Bennett 1963(154) | 329 | RCT | Meningitis cases treated with HC (n=38) ^A^ | Meningitis cases treated with placebo (n=47) | Mortality | Overall study included patients with any severe infection; no difference in mortality observed between groups | High | Imprecision  Indirectness |
| Bademosi  1979(155) | 52 | RCT | Pneumococcal meningitis treated with HC 100mg IV on admission and then prednisolone 60mg daily in divided doses enterally for at least 14 days  (n=24) | No corticosteroid (n=28) | Mortality, neurologic sequelae | No difference in outcomes of interest between groups | High | Imprecision  Indirectness |
| Girgis 1989(70) | 429 | P | DEX 12 mg IM (n=210) ^B^ | No DEX (n = 219) | Mortality, permanent neurologic sequelae | Included pediatric & adult patients; lower mortality in pneumococcal meningitis patients who received DEX (13.5% vs 40.7%, p<0.002); hearing impairment & paresis at discharge & 6-months were significantly different only in pneumococcal meningitis patients who received DEX | High | Indirectness  Imprecision |
| Gupta 1996(156) | 75 | P | DEX 8mg IV every 6 hr for 7 days (n=36) | No DEX (n=39) | Mortality, neurologic complications | No difference in mortality & neurologic complications between groups | High | Imprecision |
| Bhaumik 1998(157) | 30 | RCT | DEX 4mg IV every 6 hr for 4 days, then tapered for 7 total days  (n=14) | No DEX  (n=16) | Audiologic & neurologic sequelae | Included patients >12 years of age; no difference in audiologic & neurologic sequelae between groups | High | Imprecision |
| Thomas 1999(158) | 60 | RCT | DEX 10 mg IV every 6 hr for 3 days  (n=31) | Placebo (n=29) | Cure rate without neurologic sequelae | DEX had no impact on mortality or neurologic sequelae; study was stopped early due to updated antibiotic recommendations being released | High | Imprecision |
| Ahsan 2002(159) | 68 | P | DEX 0.6 mg/kg/day IV in 3 divided doses for 4 days (n=unclear) | No DEX (n=unclear) | Mortality, morbidity | Included patients >11 years of age; no difference in mortality or morbidity (defined as having a focal neurologic deficit) between groups | High | Imprecision  Indirectness |
| Auburtin 2002 (160) | 80 | R | DEX 0.15 mg/kg IV every 6 h for 2-4 days (n=22) | No DEX (n=58) | Mortality | DEX significantly reduced the risk of death (adjusted OR 0.069; 95% CI 0.005 to 0.9; p=0.048), which was adjusted for identified prognostic factors | High | Imprecision  Indirectness |
| de Gans 2002 (68) | 301 | RCT | DEX 10mg IV every 6 hr for 4 days (n=157)^C^ | Placebo  (n=144) | GOS, death, focal neurologic deficit, hearing loss | DEX associated with reduced risk of unfavorable outcome (8-week GOS 1-4; RR 0.59, 95% CI 0.37-0.94) & mortality (RR 0.48, 95% CI 0.24-0.96); no overall beneficial effects on neurologic sequelae & hearing loss; patients with pneumococcal meningitis had reduced risk of unfavorable outcomes in DEX group (RR 0.50, 95% CI 0.30-0.83) | Low | Imprecision |
| Gijwani 2002 (161) | 40 | RCT | DEX 0.6 mg/kg/day IV in 4 divided doses for 4 days (n = 20)^C^ | Placebo (n = 20) | Neurologic complications, hearing loss | Included patients > 10 years of age; DEX was associated with fewer neurologic complications at discharge; severe hearing loss was less common in the DEX group vs placebo | High | Imprecision  Indirectness |
| Flores-Cordero 2003 (162) | 62 | R | DEX (n = 40) | No DEX (n=24) | Mortality | Evaluated 64 total episodes of meningitis in 62 patients & DEX did not reduce mortality | High | Imprecision  Indirectness |
| Weisfelt 2006 (163) | 87 | P | DEX 10mg IV every 6 hr for 4 days  (n=46)^c^ | Placebo  (n=41) | Long-term neurologic audiologic & neuro-psychological outcomes | Follow-up study to de Gans 2012; no difference in any outcomes between patients receiving DEX & placebo | High | Indirectness  Imprecision |
| Weisfelt 2006 (164) | 112 | R | Pneumococcal meningitis treated with DEX 10 mg IV every 6 hr (n = 60) | Pneumococcal meningitis without DEX (n = 52) | Predictors of mortality, complications | Absence of DEX treatment found to be 1 of 5 independent predictors of death; patients treated with DEX were less likely to develop systemic & neurologic complications | High | Imprecision |
| Hoogman 2007 (165) | 227 | P | Pneumococcal (n =79) & meningococcal meningitis (n=76) | Healthy controls (n=72) | Various neuropsycho-logic tests | Patients who received DEX had some variability in some neuro-psychologic testing, but no overall differences | High | Imprecision  Indirectness |
| Nguyen 2007 (166) | 435 | RCT | DEX 0.4 mg/kg IV every 12 hr for 4 days  (n = 217)^C^ | Placebo  (n = 218) | Mortality, mRS, hearing loss | Included patients >14 years of age, significantly reduced risk of death at 1 month & in death or disability at 6 months in patients with confirmed bacterial meningitis | Low | Indirectness |
| Scarborough 2007 (66) | 465 | RCT | DEX 16 mg IV twice daily for 4 days (n = 233)^C^ | Placebo (n=232) | Mortality, GOS, hearing impairment | No difference in outcomes in both full cohort & subgroup with proven pneumococcal meningitis; 90% of included patients were HIV positive; | Low | Indirectness |
| Ayaz 2008 (167) | 144 | P | DEX 16mg/day IV for 3 days (n = 72)^D^ | No DEX (n = 72) | Mortality, consciousness level | Consciousness level in DEX group improved more rapidly than the comparator group (p=0.001); mortality was 9.7% in the DEX group & 16.7% in the control group (p>0.05) | High | Imprecision  Indirectness |
| Lazzarini 2008 (168) | 322 | P | Adjunctive corticosteroids (n = unclear) | No adjunctive corticosteroid (n = unclear) | Mortality, focal neurologic findings | In pneumococcal meningitis subgroup (n =133), no significant difference in death or neurologic impairment at discharge between groups | High | Imprecision  Indirectness |
| Vardakas 2009 (64) | 2125 | MA^E^ | Corticosteroid | Placebo or no corticosteroid | Mortality, hearing impairment | Included 10 RCTs, with 8 evaluating DEX & 2 HC or prednisone; DEX therapy showed no difference in all-cause mortality compared to comparator group^f^; DEX was associated with fewer episodes of hearing impairment in high quality RCTs (OR 0.64, 95% CI 0.43–0.94); lower mortality in definite meningitis with shorter symptom duration, pneumococcal meningitis, & patients in countries with high & medium Human Development Index | Low | Imprecision  Indirectness |
| Assiri 2009 (169) | 1261 | MA^G^ | DEX IV at varying doses before or within 3 hr of antibiotics (n=636) | Placebo  (n =619) | Mortality rate | Included 4 RCT of adult patients; corticosteroid use resulted in a lower short-term mortality than placebo only in high-income countries (RR 0.5, 95% CI 0.27-0.92), & in studies with a low prevalence of infection with HIV (RR 0.66, 95% CI 0.44-0.99) | Low |  |
| Tolaj 2010 (170) | 147 | RCT^H^ | Meningococcal disease & CNS infection (n=130) | Meningococcal disease & no CNS infection (n=17) | Mortality, CSF glucose & protein | Included both adult & pediatric patients (15 patients were > 12 years old); DEX did not impact mortality & CSF protein, but normalized CSF glucose earlier; 64.6% of treatment groups received DEX 0.15 mg/kg IV every 6 hr for 4 days, with 47.1% of comparator receiving the same | High | Imprecision  Indirectness |
| van de Beek 2010 (67) | 2029 | MA^I^ | DEX at varying doses  (n = 1019)^c^ | Placebo  (n = 1010) | Mortality, neurologic sequelae, GOS, mRS | Included both adult & pediatric patients in an individual patient data meta-analysis; no significant reduction in rates of death (26.5% DEX vs 27.2% placebo), neurologic disability, or severe hearing loss; no difference in outcomes in any pre-specified subgroup, including age; post-hoc analysis found that DEX reduced hearing loss among survivors (OR 0.77, 0.60–0.99) | Low |  |
| Brouwer 2010 (171) | 709 | R | Pneumococcal meningitis treated between 2006-2009 (n=357 episodes) | Historical control of pneumococcal meningitis (n=352) | GOS,  neurologic outcome | Odds of unfavorable outcome (GOS 1-4) at hospital discharge were significantly reduced in intervention group (OR 0.63, 95% CI 0.46-0.86), as were rates of death & hearing loss; differences in outcomes remained after adjusting for imbalances between cohorts; 92% of treatment group episodes received DEX at various doses (84% given with or before first antibiotic dose) compared to 17% in comparator group (3% with or before first antibiotic dose) | High | Indirectness |
| Fritz 2012 (69) | 278 | R | DEX 10mg IV every 6 hr for 4 days  (n=144) ^c^ | Placebo  (n = 134) | Long term survival | Follow-up study to de Gans 2012; 31 of 144 DEX group patients (22%) died compared to 44 of 134 placebo group patients (33%; log-rank p=0.029); beneficial effects most apparent with pneumococcal meningitis (p=0.009) | High | Imprecision |
| Heckenberg 2012 (172) | 354^j^ | R | Meningococcal meningitis treated between 2006-2011 (n=96 episodes) | Historical control of meningococcal meningitis (n=258) | Mortality, GOS, hearing loss | DEX given before or with the first dose of antibiotics in 89% of cases; rates of death, hearing loss, & unfavorable outcome (GOS 1-4) at discharge were similar; 90% of episodes in treatment group received DEX compared to 17% in comparator | High | Indirectness  Imprecision |
| Moon 2012 (173) | 93 | R | Adequate corticosteroid use (n=42) or inadequate use (n=26) in pneumococcal meningitis | No corticosteroid in pneumococcal meningitis  (n=25) | Mortality, neurologic sequelae | Adequate use defined as starting corticosteroid before or with first dose of antimicrobials; exact corticosteroid & dose not specified; 30-day mortality was no different between any group; adequate corticosteroid use did not reduce neurologic sequelae | High | Imprecision  Indirectness |
| Bodilsen 2014 (174) | 172 | R | Meningitis patients treated after guideline recommended DEX (n=97) | Historical control with no DEX  (n=75) | Mortality, GOS, auditory sequelae | No difference in outcomes of interest between study groups; 47.4% of treatment group received DEX 10 mg IV every 6 hr for 4 days | High | Imprecision  Indirectness |
| Fernandes 2014 (175) | 65 | R | Favorable clinical outcome from meningitis (n=19) | Adverse clinical outcome from meningitis (n=46) | Corticosteroid usage | No difference in corticosteroid use in favorable vs adverse clinical outcome cohorts; corticosteroid use included DEX at least 0.15mg/kg every 6 hr for at least 4 days or equivalent, started within 24 hr | High | Imprecision  Indirectness |
| Viale 2015 (176) | 177 | R | Meningitis bundle (n=85) | Historical control  (n = 92) | Mortality,  neurologic sequelae | Bundle included DEX 10mg IV followed by 0.15 mg/kg every 6 hr for 4 days; DEX use was associated with better outcome (HR 0.34, 95% CI 0.12-0.94); no difference in rate of neurologic sequelae at discharge | High | Imprecision  Indirectness |
| Brouwer 2015 (63) | 4121 | MA^K^ | Corticosteroid (n=2064) | Placebo (n=2057) | Mortality, hearing loss, neurologic sequelae | Included 25 studies of pediatric & adult patients; 22 studies included DEX & 3 included HC or PRED; 7 studies with adult patients found no difference in mortality; significantly lower rates of hearing loss in corticosteroid-treated patients (4 studies; RR 0.74, 95% CI 0.56-0.98); corticosteroid-treated patients with pneumococcal meningitis had lower mortality & lower rates of hearing loss only in high-income countries (10.1% vs 17.6%) | Low |  |
| Baunbaek-Knudsen 2016 (177) | 147 | R | DEX 10mg IV or equivalent dose of other corticosteroid every 6h for 4 days (n = 104) | No corticosteroids (n = 43) | Mortality, GOS | Corticosteroid treatment did not impact mortality, but was associated with a favorable outcome (GOS=5; RR0.48, 95% CI 0.30–0.76); 31% of patients had an immunosuppressive co-morbidity | High | Imprecision  Indirectness |
| Buchholz 2016 (178) | 142 | R | Pneumococcal meningitis treated between 2003-2015 (n=55) | Historical control of pneumococcal meningitis (n=87) | GOS, mortality | Lack of DEX was associated with death in all patients in both study periods, but not with an unfavorable outcome based upon GOS; 85.5% of treatment group episodes received DEX at varying doses compared to 18.4% of comparator group; no patients received DEX before first antibiotic dose | High | Indirectness  Imprecision |
| Gudina 2018 (179) | 90 | P | DEX (n = 30) | No DEX  (n = 60) | Factors associated with unfavorable outcome | DEX therapy found to be one of three factors independently associated with unfavorable outcome; DEX dosing not specified | High | Indirectness  Imprecision |
| ^A^ Hydrocortisone 300mg IV over 6-8 hr day 1, 250mg IV over 6-8 hr day 2; Hydrocortisone tablets orally 200 mg given on the third day, 150 mg on the fourth, & decreasing by 50 mg per day for total of 6 days of hydrocortisone; ^B^ Dosing of DEX IM every 12 hr for 3 days for adults & 8 mg intramuscularly every 12 hr for 3 days for children < 12 years old; ^C^ Study protocol stated that dexamethasone was given at least 15-20 min before or with first dose of antibiotics; ^D^ Dosing of DEX listed as “given 10-15 min before the first 8 mg dose of antibiotic treatment. It was continued at 16 mg/day for 3 days.” Reviews interpreted this to mean that the 8 mg dose of DEX was given 10-15 min before the first antibiotic dose; ^E^ Meta-analysis included studies by Girgis 1989, Gupta 1998, Thomas 1999, Gijwani 2002, de Gans 2002, Nguyen 2007, Scarborough 2007; ^F^ When Scarborough 2007 was excluded, DEX was associated with lower mortality (OR = 0.58, 95% CI 0.40-0.83); ^G^ Meta-analysis included studies by Thomas 1999, de Gans 2002, Scarborough 2007, Nguyen 2007; ^H^ Dexamethasone dosing listed as 0.15mg/kg IV every 6 hr for 4 days with first dose given up to 30 min before antibiotics in both groups;  ^I^ Meta-analysis included studies by Nguyen 2007, Scarborough 2007, de Gans 2002; ^J^ Study lists 358 total patients, but outcome data related to DEX use only reported on 354 per Table 2; ^K^ Meta-analysis included studies by Bennett 1963, de Gans 2002, Girgis 1989, Nguyen 2007, Scarborough 2007, Thomas 1999, Bhaumik 1998  The following studies (with reasoning) were excluded from our assessment: Martino 2018 (meta-analysis evaluating corticosteroid use in critically ill patients also included a subgroup assessment of bacterial meningitis; evaluation of included studies revealed that a study evaluating TB meningitis was also included in this assessment; reviewers contacted the corresponding author of the paper who supplied unpublished data demonstrating no difference in reported outcomes when only the studies of bacterial meningitis were included, which differed than results published in original manuscript) | | | | | | | | |

Risk of Bias Table 8. Studies evaluating the use of adjunctive corticosteroid therapy in tuberculous meningitis

| Reference | Patient Number | Study Design | Intervention | Comparator | End points of interest | Findings | Risk of bias (high/low/unclear) | Comments on risk of bias |
| --- | --- | --- | --- | --- | --- | --- | --- | --- |
| O’Toole 1969 (180) | 23 | RCT | DEX (n = 11)^A^ | No DEX (n=12) | Mortality,  opening pressure | Included both adult & pediatric patients; no difference in mortality with opening pressure reduced over time in DEX group | High | Imprecision  Indirectness |
| Girgis 1991 (181) | 160 | RCT | DEX 12mg/day IM to adults (8 mg/day IM if weight <25 kg) for 3 weeks (n=75) | No DEX (n=85) | Mortality; physical, neurologic & ophthal-mologic exams | Included both adult & pediatric patients; 160 confirmed TB cases; mortality rate (43% vs 59%) & neurologic complications were significantly lower in the DEX group | High | Indirectness  Imprecision |
| Kumarvelu 1994 (79) | 47 | RCT | DEX (n =24)^B^ | No DEX (n=23) | Neurologic examination, mini-mental score, ADL, auditory function | Included both pediatric & adult patients; no difference in any outcomes of interest between groups | High | Imprecision  Indirectness |
| Chotmongkol 1996 (78) | 59 | RCT | PRED oral (n=29)^C^ | Placebo (n =30) | Mortality, focal neurologic abnormalities | Included patients > 15 years of age; no significant difference in outcomes between groups | High | Indirectness  Imprecision |
| Yechoor 1996 (182) | 31 | R | Corticosteroids  (n = 12) | No corticosteroid  (n = 17) | Mortality | Steroids were given in non-systematic manner with medication & dose not reported; no difference observed in 9-month mortality | High | Imprecision  Indirectness |
| Kalita 2001 (183) | 37 | P | MP 500mg IV daily for 5 days, followed by PRED oral taper over 1 month  (n = 21) | No corticosteroid  (n = 16) | MEP, SEP | No difference in outcomes between cohorts | High | Imprecision  Indirectness |
| Thwaites 2004 (77) | 545 | RCT | DEX (n = 274)^D^ | Placebo  (n = 271) | Mortality, disability | Included patients >14 years of age; DEX reduced risk of death, but not severe disability; effects were consistent across subgroups of disease-severity grade & 98 HIV patients | Low |  |
| Malhotra 2009 (80) | 91 | RCT | DEX for 8 weeks (n = 31)  or MP for 5 days (n = 30)^E^ | No corticosteroid (n=30) | Mortality, functional outcome. focal neurologic findings | Corticosteroid use associated with reduced death or disability, but not statistically significant when agents analyzed individually; rate of vision impairment among survivors decreased from 41.8% at baseline to 29.9% at 6 months | High | Indirectness |
| Rahman 2009 (184) | 13 | P | DEX IV for 4 weeks (n = 8)^F^ | PRED 1 mg/kg orally x 4 weeks then taped over 4 weeks  (n = 5) | Mortality, disability | All DEX patients survived, but 7 were discharged with disease stigma; 3 of 5 PRED patients died; disability assessment limited due to only 4 patients coming for follow-up | High | Imprecision  Indirectness |
| Hsu 2010 (185) | 108 | R | “Definite” meningitis (n = 46) | “Probable” meningitis (n = 62) | Mortality | Adjunctive corticosteroid use defined as PRED ≥ 20mg/day for at least 7 days; patients receiving corticosteroids for over 2 & 3 weeks had increased number of survivors in definite TB & overall cohorts; receiving steroids for over 2 weeks increased survivors in probable TB | High | Imprecision  Indirectness |
| Torok 2011 (186) | 545 | P | DEX  (n=274)^D^ | Placebo  (n = 271) | Long-term survival, disability status | Five-year survival rates were similar in both groups; similar proportion of severely disabled patients at 5 years | High | Imprecision  Indirectness |
| Prasad 2016 (76) | 1337 | MA^G^ | Corticosteroid  (n = 688) | Placebo or no treatment  (n = 649) | Death, neurologic deficit | Included both pediatric & adult studies; corticosteroids (HC, PRED, MP, or DEX) reduced deaths (RR 0.75, 95% CI 0.65-0.87); no difference in disabling neurologic deficits | Low | Indirectness  Inconsistency |
| ^A^ DEX IV given every 6 hr at the following doses for adults: 2.25 mg for the first week, followed by 1.5mg for the second week, followed by 0.75 mg during week 3, then 0.375 mg during week 4; ^B^ DEX 16mg IV in 4 divided doses for 7 days, followed by DEX 8 mg/day orally as a single dose for 21 days; ^C^ PRED 20 mg orally three times a day for 1 week; then, dose decreased to 45, 30, 20, & 10mg per day for 1 week each; ^D^ For grade II or III disease, DEX started at 0.4 mg/kg/day IV for week 1, then decreased by 0.1 mg/kg/day each week for 4 weeks; then, oral treatment started at a total of 4 mg/day & decreasing by 1 mg each week; For grade I disease, DEX started at 0.3 mg/kg/day IV for week 1 & 0.2mg/kg/day for week 2; then, oral therapy started at 0.1 mg/kg/day for one week, decreasing to a total of 3 mg/day & decreasing by 1 mg each week; ^E^ DEX IV dosed at 0.4, 0.3, 0.2, 0.1 mg/kg/day each week for 4 weeks; then, oral treatment started at 4 mg/day & decreasing by 1 mg/day each week; MP IV dosing for 5 days at 1g for patients weighing > 50 kg or 20 mg/kg if < 50 kg; ^F^ DEX dosed at 0.4 mg/kg/day for 1 week, 0.3 mg/kg/day x 1 week, then 0.2 mg/kg/day x 1 week, then 0.1 mg/kg/day x 1 week; then oral treatment x 4 weeks starting at 4 mg per day & decreasing by 1 mg each week; ^G^ Meta-analysis included studies by Chotmongkol 1996, Girgis 1991, Kumarvelu 1994, Malhotra 2009, O’Toole 1969, & Thwaites 1994 | | | | | | | | |

Risk of Bias Table 9. Studies evaluating use of hypertonic sodium solutions & mannitol in patients with bacterial meningitis

| Reference | Patient Number | Study Design | Intervention | Comparator | End points of interest | Findings | Risk of bias (high/low/unclear) | Comments on risk of bias |
| --- | --- | --- | --- | --- | --- | --- | --- | --- |
| Glimaker 2014 (72) | 105 | P | ICP-targeted treatment (included hypertonic NaCl [n=21] & rescue mannitol [n=5]) (n=52) | Controls without ICP-targeted therapy (n=53) | Mortality, GOS | Mortality was significantly lower in intervention group compared to comparator (10% vs 30%; p<0.05); full recovery (GOS 5 with normal hearing) was higher in the intervention group (54% vs 32%; p<0.05) | High | Imprecision |

Risk of Bias Table 10. Studies evaluating use of hypertonic sodium solutions & mannitol in patients with hepatic encephalopathy

| Reference | Patient Number | Study Design | Intervention | Comparator | End points of interest | Findings | Risk of bias (high/low/unclear) | Comments on risk of bias |
| --- | --- | --- | --- | --- | --- | --- | --- | --- |
| Hanid 1980 (83) | 10 | P | DEX 32 mg IV initially, then 8 mg every 4 hr plus mannitol 40 -100 g if needed (n=6) | DEX 32 mg IV initially, then 8 mg every 4 hr (n=4) | ICP | Total of 6 patients received mannitol, which consistently arrested ICP elevation when ICP was < 60 mmHg; continued increases in ICP were observed in majority of patients despite use of DEX | High | Indirectness  Imprecision |
| Canalese 1982 (81) | 44 | RCT | Group 1: DEX 32 mg IV initially, then 8 mg every 6 hr (n=11)  Group 2: mannitol 20% 1 g/kg (n=10) | Group 3: DEX IV & mannitol at same doses as groups 1 & 2 (n=10)  Group 4: neither DEX or mannitol (n=13) | ICP, cerebral edema | In 9 patients with an ICP monitor who received mannitol, ICP fell by a mean of 22.6 mmHg; in 8 patients without ICP monitoring who developed clinical deterioration, mannitol use demonstrated improvement in 17 of 20 patients’ neurologic examination; no difference in cerebral edema development with addition of DEX | High | Indirectness  Imprecision |
| Murphy 2004 (82) | 30 | RCT | 30% NaCl infusion with goal Na 145-155 mEq/L (n=15) | Standard of care (n=15) | ICP | ICP decreased significantly from baseline over the first 24 hr in the intervention group; rates of intracranial hypertension significantly higher in comparator group | High | Indirectness  Imprecision |
| Saraswat 2008 (84) | 10 | P | 20% Mannitol 1g/kg infusion (n=5) | Healthy controls (n=5) | Brain water content, neurologic examination | Mannitol did not significantly reduce brain water content or change neurologic examination | High | Indirectness  Imprecision |
| Liotta 2015 (85) | 19 | R | 23.4% NaCl 30 mL (n=11) | No 23.4% NaCl (n=8) | Brain tissue volume on CT, GCS | No reduction in brain tissue volume, although GCS scores improved in patients receiving 23.4% NaCl | High | Indirectness  Imprecision |

Risk of Bias Table 11. Studies evaluating use of osmolar gap & osmolarity in patients receiving mannitol

| Reference | Patient Number | Study Design | Intervention | Comparator | End points of interest | Findings | Risk of bias (high/low/unclear) | Comments on risk of bias |
| --- | --- | --- | --- | --- | --- | --- | --- | --- |
| Dorman 1990 (86) | 8 NCC | CS | Mannitol 20% (mean daily dose 189 + 64g) | None | AKI | Peak osmolar gap (74 + 39 mOsm/kg) correlated significantly with AKI development occurring within 3.5 + 1.5 days & lasting for 7-10 days after stopping mannitol | High | Indirectness  Imprecision |
| Dziedzic 2003 (87) | 51 ICH | CS | Mannitol 20% 0.25-0.5 g/kg every 4 hr & furosemide 10 mg every 8 hr for up to 5 days | None | Serum osmolarity,  Urea concentration | Mannitol held if osmolarity > 310 mOsm/L; no patients developed anuria or oliguria after mannitol given; relationship observed between creatinine & serum osmolarity, but not urea level & serum osmolarity | High | Imprecision  Indirectness |
| Gondim 2005 (88) | 95 NCC | R | Mannitol 20% | None | AKI | Mannitol held only if osmolar gap began to double or increased above 20 mOsm/kg; 11.6% had AKI; renal function returned to baseline within 15 days without hemodialysis; osmolar gap was not one of the two variables (CHF, APACHE II score) identified on multivariable analysis to be independently associated with AKI | High | Indirectness  Imprecision |

Risk of Bias Table 12. Studies evaluating adverse events associated with hypertonic sodium solutions administration

| Reference | Patient Number | Study Design | Intervention | Comparator | End points of interest | Findings | Risk of bias (high/low/unclear) | Comments on risk of bias |
| --- | --- | --- | --- | --- | --- | --- | --- | --- |
| Suarez 1999(107) | 29 SAH | R | 3% NaCl / Na Acetate infusion 100-200 mL/hr | None | Serum Na & chloride, metabolic acidosis | Intervention administered only to patients with serum Na < 135 mEq/L; Significant increase in serum Na &Cl without increased rate of metabolic acidosis | High | Indirectness  Imprecision |
| Munar 2000 (138) | 14 TBI | P | 7.2% NaCl 1.5mL/kg  (n=14) | None | Serum Na & chloride, SCr | No change in SCr at any interval up to 2 hr administering 7.2% NaCl; Serum Na & chloride significantly increased during the 2 hr period | High | Imprecision |
| Larive 2004 (101) | 19 NCC | R | 2-3% NaCl / Na Acetate infusion with goal Na 145-155 mEq/L  (n = 19) | None | Adverse events | Adverse events (including, but not limited to: AKI, metabolic acidosis or alkalosis, electrolyte abnormalities) were not significantly different between groups | High | Indirectness  Imprecision |
| Ware 2005 (22) | 13 TBI | R | 23.4% NaCl 30mL (n=22 doses) | Mannitol bolus in varying doses (mean 0.86 g/kg)  (n=19 doses) | Serum osmolarity, adverse events | All patients received both interventions; no adverse events associated with 23.4% NaCl with average Na increase after 23.4% NaCl being 5 mEq/L & 1 mEq/L after mannitol | High | Indirectness Imprecision |
| Tseng 2007 (16) | 35 SAH | P | 23.5% NaCl 2ml/kg with goal Na 145-155 mEq/L | None | Serum electrolytes, arterial bicarbonate, renal function parameters | Na & osmolarity increased maximally at 1 hr by 11.23 ± 4.05 mEq/L & 23.21 ± 11.85 mOsm/L; hypernatremia & hyperosmotic effects persisted longer than 24 hr; levels of other serum electrolytes, arterial bicarbonate, & renal function were unchanged | High | Imprecision  Indirectness |
| Froelich  2009 (99) | 187 NCC | R | 2% or 3% NaCl continuous infusion  (n=107) | 0.9% NaCl continuous infusion (n=80) | Na, renal function parameters, infection, VTE, dural sinus thrombosis | Rates of moderate (Na >155 mEq/L) & severe hypernatremia (Na >160 mEq/L) were significantly higher in continuous infusion group; no significant relationship between 2-3% NaCl & renal dysfunction, although serum Na above 155 mEq/L was associated with renal dysfunction; no difference in other outcomes | High | Indirectness |
| Oddo 2009 (102) | 12 TBI | R^B^ | Mannitol 25% 0.75g/kg  (n=28 doses) | 7.5% NaCl 250 mL  (n=14 doses) | Serum Na, osmolarity, adverse events | Treatment with 7.5% NaCl was associated with significantly higher serum Na, but not osmolarity; hypernatremia (serum Na >155 mEq/L) was observed in three patients receiving 7.5% NaCl; no other adverse events, including AKI, were observed | High | Indirectness Imprecision |
| Kerwin 2009 (103) | 22 TBI | R | 23.4% NaCl 30mL  (n=108 doses) | Mannitol 15g - 75g  (n=102 doses) | Serum Na, chloride, renal function parameters | Maximum increase of Na was 11 mEq/L & maximum increase of serum osmolality was 27 mOsm/L; SCr elevation observed only in 4 patients, all of which died | High | Indirectness Imprecision |
| Hauer 2011 (9) | 215 NCC | R | 3% NaCl infusion with goal Na 145-155 mEq/L  (n=100) | Historical cohort not receiving 3% NaCl (n=115) | Adverse events | Adverse events (including cardiac arrhythmia, heart, liver or renal dysfunction, pulmonary edema) occurred similarly in both groups | High | Imprecision  Indirectness |
| Corry 2014 (105) | 230 NCC | R | 23.4% NaCl, 3% NaCl, 3% NaCl / Na Acetate (n=35) | 0.9% NaCl or LR (n=194) | Renal function parameters | No significant correlation occurred with Na or chloride with SCr when grouped according to fluid type | High | Indirectness |
| Ong 2015 (43) | 30 AIS | R | Medical management success (n=14) | Medical management failure (n=16) | Adverse events | Success group included hyperosmolar therapy (mannitol &/or 23.4% NaCl) & hospital survival, while failure group included hyperosmolar therapy & surgery or death; peak hypernatremia (> 160mEq/L) occurred in 14% of patients with success compared to 38% in failure patients; high rates of AKI in both groups | High | Indirectness  Imprecision |
| Jagannatha 2016 (30) | 38 TBI | RCT | 3% NaCl 2.5ml/kg  (n=18) | Mannitol 20% 2.5ml/kg  (n=20) | Serum Na, osmolarity, renal function parameters | Serum Na, osmolality & renal function parameters were comparable between groups | Low | Imprecision  Indirectness |
| Sadan 2017 (104) | 1267 SAH | R | AKI (n=212) | Non-AKI (n=1055) | Mean daily serum Na, chloride, renal function parameters | Treatment with hypertonic NaCl was more common in the AKI group; treatment with hypertonic NaCl & increase in mean serum chloride were found to independently predict AKI development | Low^A^ |  |
| Erdman 2017 (100) | 337 NCC | R | 3% NaCl continuous infusion (n=337) | None | Renal function parameters | 16% of patients developed AKI, AKI associated with longer LOS & higher mortality (48.1% vs 21.9); Hyperchloremia (Cl > 110 mEq/L), severe hypernatremia (Na > 155 mEq/L), & hyperosmolality were more common in the AKI group; serum Na > 155 mEq/L was found to independently predict AKI development | High | Lack of comparator group, imprecision |
| Maguigan  2017 (98) | 162 TBI | R | 3% NaCl continuous infusion (n=132) | 3% NaCl bolus dosing (n=30) | Electrolyte values, renal function parameters | Similar rates of hypernatremia, but significantly higher rates of hyperchloremia & AKI (0% vs 12.9%) observed in patients treated with 3% NaCl continuous infusion | High | Indirectness |
| Riha 2017 (106) | 100 ICH | R | 3% NaCl continuous infusion  (n=100) | None | Renal function parameters | Propensity-matched cohort divided patients into those who developed hyperchloremia & those who did not; rates of AKI were significantly higher in those with hyperchloremia | High | Indirectness |
| ^A^ While risk of bias was possibly present, it was not severe enough to warrant downgrading of evidence; ^B^ Rescue therapy with either agent was allowed by crossover when ICP not controlled | | | | | | | | |

Risk of Bias Table 13. Studies comparing continuous infusion & bolus administration of hypertonic sodium solutions in patients with cerebral edema

| Reference | Patient Number | Study Design | Intervention | Comparator | End points of interest | Findings | Risk of bias (high/low/unclear) | Comments on risk of bias |
| --- | --- | --- | --- | --- | --- | --- | --- | --- |
| Maguigan  2017 (98) | 162 TBI | R | 3% NaCl continuous infusion (n=132) | 3% NaCl bolus dosing (n=30) | ICP, CPP, mortality | No differences in CPP, ICP or mortality observed between groups | High | Imprecision |
| The following studies that described a single administration method for hypertonic sodium solutions were excluded from our assessment because they did not meet the predetermined specifications set forth by the PICO question: Qureshi 1999, Froelich 2009, Roquilly 2011, Hauer 2011, Wagner 2011, & Li 2015. | | | | | | | | |

Risk of Bias Table 14. Studies evaluating the effect of head of bed positioning, hyperventilation, & cerebrospinal fluid diversion on cerebral edema

| Reference | Patient Number | Study Design | Intervention | Comparator | End points of interest | Findings | Risk of bias (high/low/unclear) | Comments on risk of bias |
| --- | --- | --- | --- | --- | --- | --- | --- | --- |
| Kenning 1981 (110) | 24 NCC | P | HOB elevated to 45° & 90° | None (subjects served as own control) | ICP | Regardless of the initial supine position value, ICP was reduced at 45° & 90°; 13 patients had documented intracranial hypertension | High | Imprecision  Indirectness |
| Durward 1983 (109) | 11 TBI | P | HOB positioned to 0°, 15°, 30° & 60° | None (subjects served as own control) | ICP, CPP | 15° or 30° HOB elevation provided a consistent decrease in ICP; 0° or 60° may be detrimental to the patient because of changes in ICP & CPP | High | Imprecision  Indirectness |
| Rosner 1986 (111) | 18 NCC | P | HOB elevated from 0° to 50° in 10° increments | None (subjects served as own control) | ICP, CPP | Average ICP decreased by 1mmHg for every 10° raised, but this was associated with an average decrease in CPP by 2-3mmHg due to concomitant decrease in SBP with HOB elevation; maximal CPP was always in the horizontal position | High | Imprecision |
| Feldman 1992 (112) | 22 NCC | P | HOB elevated from 0° to 30° | None (subjects served as own control) | ICP, CPP, CBF | Mean ICP was significantly lower when the patient's HOB was elevated at 30° than at 0° (14.1 + 6.7 mmHg vs. 19.7 + 8.3 mmHg); no statistically significant change in any other outcome with HOB position change | High | Imprecision |
| Moraine 2000 (113) | 37 NCC | P | HOB elevated to 0°, 15°, 30°, 45° | None (subjects served as own control) | ICP, CPP | ICP decreased between 0° & 30° HOB elevation, but increased between 30° & 45°; CPP remained stable between 0° & 30° HOB elevation, but decreased between 30° & 45° | High | Indirectness |
| Winkelman 2000 (114) | 8 TBI | P | HOB elevated to 30° | None (subjects served as own control) | ICP, CPP | Improvements in both ICP & CPP observed with 30° HOB elevation; subjects experienced a mean ICP decrease of 4.0 mmHg when HOB was elevated from 0 to 30° | High | Imprecision  Indirectness |
| Ng 2004 (117) | 38 TBI | P | HOB started at 30°& lowered to 0° | None (subjects served as own control) | ICP, CPP, MAP | ICP was significantly lower at 30° than at 0° HOB elevation; MAP unchanged & CPP was slightly higher at 30°; global venous cerebral oxygenation & regional cerebral oxygenation were not affected significantly by HOB elevation | High | Imprecision Indirectness |
| Mahfoud 2010 (116) | 33 NCC | P | HOB elevated to 0°, 30°, 60° | None (subjects served as own control) | ICP, CPP, intracranial pulse pressure | When HOB elevations changed from 0° to 60°, there was a significant improvement in ICP, increase in intracranial pulse pressure, reduction in MAP & decrease in CPP; ICP also decreased with 0° to 30° change | High | Imprecision  Indirectness |
| Ledwith 2010 (115) | 30 NCC | P | Each patient placed in 4 body positions including 3 HOB angles (15°, 30°, 45°) | None (subjects served as own control) | ICP, CPP | Body positions included: supine, supine with knee bent, left lateral position, right lateral; ICP decreased with supine HOB 45° & knee elevation, as well as HOB 30° & 45°, & increased with right & left lateral HOB 15° | High | Imprecision |
| Muizelaar 1991 (121) | 113 TBI | RCT | HPV (n=36) &  HPV plus tromethamine (n=36) | Normal ventilation (n=41) | ICP, GOS | HPV defined as PaCO_2_ 25 + 2 mmHg; patients treated with HPV & tromethamine had the most stable ICP course, but this did not significantly impact GOS scores | High | Imprecision  Indirectness^A^ |
| Fortune 1995 (119) | 22 TBI | P | HPV, mannitol 25 g IV bolus, or CSF drainage at discretion of treating physician | None | ICP, CBF | 196 elevated ICP episodes occurred with CSF drainage used in 67%, mannitol in 25%, & HPV in 8%; after CSF drainage, ICP fell in 90% of episodes by 8.6 mmHg; after HPV, ICP fell in 88% of observations by 6.3 mmHg; after mannitol, ICP fell in 90% of observations by 7.4 mmHg; mannitol resulted in the most improvement in CBF between the 3 strategies | High | Lack of comparator, Imprecision  Indirectness |
| Oertel 2002(118) | 33 TBI (27 with HPV) | P | HPV, metabolic suppression, induced hypertension | None | ICP | HPV reduced PaCO2 by 8mmHg; ICP was reduced in 96.5% of events, mean decrease was 37% from baseline | High | Indirectness |
| Soustiel 2006 (120) | 36 TBI | P | HPV  combined with mannitol 20% 0.5 g/kg | Baseline data before intervention | CBF, ICP, CPP | HPV defined as reducing PaCO2 from 36 + 4 to 32 + 4mmHg, which did not produce a significant ICP change; CPP increased by 5.6% with a statistically significant CBF decrease | High | Imprecision  Indirectness |
| Kerr 2000 (122) | 36 TBI | P | CSF drainage^B^ | None | ICP | After CSF drainage, there was a significant mean change in ICP of 4 mmHg; one-third of patients experienced a decrease in ICP below 20 mmHg | High | Inconsistent  Indirectness |
| Nwachuku 2013 (125) | 62 TBI | R | Intermittent CSF drainage (n=31) | Historical matched cohort with closed EVD (n=31) | ICP | Compared patients with closed EVD with intermittent CSF drainage versus open EVD with continuous drainage; Mean ICP was 5.66 mmHg higher (p < 0.0001) in the closed EVD group; ICP burden > 20 mmHg was significantly higher in the closed EVD group (p = 0.0002) | High | Indirectness  Imprecision |
| Kerr 2001 (123) | 58 TBI | P | CSF drainage^B^ | None | ICP, CPP | Significant dose-time interaction for ICP with the three-extraction volume protocol with incremental decreases in ICP; 3-ml withdrawal of CSF resulted in a 10.1% decrease in ICP & a 2.2% increase in CPP, which were sustained for 10 minutes | High | Inconsistent  Indirectness |
| Candanedo 2018 (124) | 60 NCC | P | CSF drainage | None | Pressure equalization ratio, ICP, volume of CSF drained | TBI patients had significantly higher pre-drainage ICP (26 ± 10 mmHg) than non-TBI patients (19 ± 5 mmHg) & also drained less CSF; pressure equalization ratio was substantially higher in non-TBI | High | Indirectness |
| ^A^ Very serious concerns of indirectness resulted in downgrading of evidence by two levels; ^B^ Patients underwent three randomly ordered CSF drainage protocols that varied in the volume removed (1 mL, 2 mL, & 3 mL) for a total of 6 mL of cerebrospinal fluid removed | | | | | | | | |
